# Supplementary material for: The natural history of ataxia-telangiectasia (A-T): A systematic review
Source: PLoS One. 2022 Mar 15;17(3):e0264177. doi: 10.1371/journal.pone.0264177 (PMC9049793; doi:10.1371/journal.pone.0264177)
Supplement: S1 Protocol — (PDF) [file pone.0264177.s002.pdf]

Summary of search protocol

Category	Search terms
Natural history	‘natural history’, pattern
Diagnosis 1	diagnosis, presentation
Diagnosis 2	AFP, alpha-fetoprotein, ‘alpha fetoprotein’, age, delay
Respiratory	respirat*, ‘sinopulmonary disease’, Bronchi*, ‘interstitial lung disease’, ILD, ‘lung disease’
Neurology	neurol*, neuroimag*, neurodisabil*, ‘nerv* system, ‘oculomotor apraxia’, cerebell*, ataxia, dysarthria*, ‘movement disorder’, CT, ‘computed tomography’, MRI, ‘magnetic resonance imaging’, bradykinesia, dystonia, myoclonus, choreoathetosis, neurodegener*, tremor, PET, ‘positron emission tomography’
Gastrointestinal	growth, nutrition, malnutrition, aspiration, dysphagia, PEG, gastroin*, gastrostomy, ‘percutaneous endoscopic gastrostomy’
Malignancy	malignancy, cancer, leukaemia, leukemia, tumour, tumor, carcinoma, neoplasm
Genetics	genot*, phenot*, genet*, *ATM*, ‘ataxia-telangiectasia mutated’, ‘ataxia-telangectasia mutated’, ‘ataxia telangiectasia mutated’, ‘ataxia telangectasia mutated’, variant
Immunology	immunol*, ‘immune system’, infection, lymphop*, lymphc*
Endocrinology	endocrine*, hormone*, diabetes, T1DM, T2DM, ‘insulin resistance’, ‘vit* D’ or ‘vit* E’
Orthopaedics	‘skeletal abnormalit*’, scoliosis, ‘foot deformit*’, fracture, bone
Dermatology	dermatology, skin, hyperpiment*, ‘café au lait’, hypopigment*, telangiectasia, telangiectasia
Death	death, mortality, survival
Pregnancy	fertility, pregnancy, childbirth
Cognition	neuropsychometric, IQ, ‘cognitive function’, intel*
Psychiatry	‘mental state’, mood, anxiety, depression
Lifestyle	‘quality of life’, lifestyle,

Search Protocol

Search as per the search terms chart. Each column needs to be searched with the first column (Ataxia-telangiectasia) using ‘AND’.

All date ranges, ages and languages.

Searching title, keywords and MeSH terms in each database.

1. PubMed

Human only filter.

- (("Ataxia-telangiectasia"[Text Word] OR "Ataxia-telangectasia"[Text Word] OR "Ataxia telangiectasia"[Text Word] OR "Ataxia telangectasia"[Text Word] OR “Louis-Bar”[Text Word] OR “Louis Bar”[Text Word])) AND (“natural history”[Text Word] OR pattern[Text Word])
- (("Ataxia-telangiectasia"[Text Word] OR "Ataxia-telangectasia"[Text Word] OR "Ataxia telangiectasia"[Text Word] OR "Ataxia telangectasia"[Text Word] OR “Louis-Bar”[Text Word] OR “Louis Bar”[Text Word])) AND (diagnosis[Text Word] OR presentation[Text Word])
- (("Ataxia-telangiectasia"[Text Word] OR "Ataxia-telangectasia"[Text Word] OR "Ataxia telangiectasia"[Text Word] OR "Ataxia telangectasia"[Text Word] OR “Louis-Bar”[Text Word] OR “Louis Bar”[Text Word])) AND (AFP[Text Word] OR “alpha-fetoprotein”[Text Word] OR “alpha fetoprotein”[Text Word] OR age[Text Word] OR delay[Text Word])
- (("Ataxia-telangiectasia"[Text Word] OR "Ataxia-telangectasia"[Text Word] OR "Ataxia telangiectasia"[Text Word] OR "Ataxia telangectasia"[Text Word] OR “Louis-Bar”[Text Word] OR “Louis Bar”[Text Word]))AND (Respirat*[Text Word] OR sinopulmonary disease[Text Word] OR Bronchi*[Text Word] OR interstitial lung disease[Text Word] OR ILD[Text Word] OR lung disease[Text Word])

- (("Ataxia-telangiectasia"[Text Word] OR "Ataxia-telangectasia"[Text Word] OR "Ataxia telangiectasia"[Text Word] OR "Ataxia telangectasia"[Text Word] OR “Louis-Bar”[Text Word] OR “Louis Bar”[Text Word]))AND (Neurol*[Text Word] OR Neuroimag*[Text Word] OR neurodisabil*[Text Word] OR "nerv* system"[Text Word] OR “oculomotor apraxia”[Text Word] OR cerebell*[Text Word] OR “ataxia”[Text Word] OR dysarthri*[Text Word] OR "movement disorder"[Text Word] OR CT[Text Word] OR “computed tomography"[Text Word] OR MRI[Text Word] OR "magnetic resonance imaging"[Text Word] OR bradykinesia[Text Word] OR dystonia[Text Word] OR myoclonus[Text Word] OR choreoathetosis[Text Word] OR neurodegener*[Text Word] OR tremor[Text Word] OR PET[Text Word] OR “positron emission tomography”[Text Word])
- (("Ataxia-telangiectasia"[Text Word] OR "Ataxia-telangectasia"[Text Word] OR "Ataxia telangiectasia"[Text Word] OR "Ataxia telangectasia"[Text Word] OR “Louis-Bar”[Text Word] OR “Louis Bar”[Text Word]))AND (Growth[Text Word] OR Nutrition[Text Word] OR malnutrition[Text Word] OR aspiration[Text Word] OR dysphagia[Text Word] OR PEG[Text Word] OR gastroin*[Text Word] OR gastrostomy[Text Word] OR ”percutaneous endoscopic gastrostomy”[Text Word])
- (("Ataxia-telangiectasia"[Text Word] OR "Ataxia-telangectasia"[Text Word] OR "Ataxia telangiectasia"[Text Word] OR "Ataxia telangectasia"[Text Word] OR “Louis-Bar”[Text Word] OR “Louis Bar”[Text Word])) AND (Malignancy[Text Word] OR cancer[Text Word] OR leukaemia[Text Word] OR leukemia[Text Word] OR tumour[Text Word] OR tumor[Text Word] OR carcinoma[Text Word] OR neoplasm[Text Word])
- (("Ataxia-telangiectasia"[Text Word] OR "Ataxia-telangectasia"[Text Word] OR "Ataxia telangiectasia"[Text Word] OR "Ataxia telangectasia"[Text Word] OR “Louis-Bar”[Text Word] OR “Louis Bar”[Text Word])) AND (Genot*[Text Word] OR phenot*[Text Word] OR genet*[Text Word] OR ATM[Text Word] OR “Ataxia-

telangiectasia mutated "[Text Word] OR "Ataxia-telangectasia mutated "[Text Word] OR "Ataxia telangiectasia mutated "[Text Word] OR "Ataxia telangectasia mutated "[Text Word] OR variant[Text Word])

- (("Ataxia-telangiectasia"[Text Word] OR "Ataxia-telangectasia"[Text Word] OR "Ataxia telangiectasia"[Text Word] OR "Ataxia telangectasia"[Text Word] OR “Louis-Bar”[Text Word] OR “Louis Bar”[Text Word])) AND (Immunol*[Text Word] OR immune system[Text Word] OR infection[Text Word] OR lymphop*[Text Word] OR lymphoc*[Text Word])
- (("Ataxia-telangiectasia"[Text Word] OR "Ataxia-telangectasia"[Text Word] OR "Ataxia telangiectasia"[Text Word] OR "Ataxia telangectasia"[Text Word] OR “Louis-Bar”[Text Word] OR “Louis Bar”[Text Word])) AND (endocrin*[Text Word] OR hormone*[Text Word] OR diabetes[Text Word] OR T1DM[Text Word] OR T2DM[Text Word] OR “insulin resistance”[Text Word] OR “vit* D”[Text Word] OR “vit* E”[Text Word])
- (("Ataxia-telangiectasia"[Text Word] OR "Ataxia-telangectasia"[Text Word] OR "Ataxia telangiectasia"[Text Word] OR "Ataxia telangectasia"[Text Word] OR “Louis-Bar”[Text Word] OR “Louis Bar”[Text Word])) AND (“skeletal abnormalit*”[Text Word] OR scoliosis[Text Word] OR “foot deformit*”[Text Word] OR fracture[Text Word] OR bone[Text Word])
- (("Ataxia-telangiectasia"[Text Word] OR "Ataxia-telangectasia"[Text Word] OR "Ataxia telangiectasia"[Text Word] OR "Ataxia telangectasia"[Text Word] OR “Louis-Bar”[Text Word] OR “Louis Bar”[Text Word])) AND (dermatology[Text Word] OR skin[Text Word] OR hyperpigment*[Text Word] OR “café au lait”[Text Word] OR hypopigment*[Text Word] OR telangiectasia[Text Word] OR telangectasia[Text Word])
- (("Ataxia-telangiectasia"[Text Word] OR "Ataxia-telangectasia"[Text Word] OR "Ataxia telangiectasia"[Text Word] OR "Ataxia telangectasia"[Text Word] OR “Louis-

Bar"[Text Word] OR "Louis Bar"[Text Word])) AND (death[Text Word] OR mortality[Text Word] OR survival[Text Word])

- (("Ataxia-telangiectasia"[Text Word] OR "Ataxia-telangectasia"[Text Word] OR "Ataxia telangiectasia"[Text Word] OR "Ataxia telangectasia"[Text Word] OR "Louis-Bar"[Text Word] OR "Louis Bar"[Text Word])) AND (fertility[Text Word] OR pregnancy[Text Word] OR childbirth[Text Word])
- (("Ataxia-telangiectasia"[Text Word] OR "Ataxia-telangectasia"[Text Word] OR "Ataxia telangiectasia"[Text Word] OR "Ataxia telangectasia"[Text Word] OR "Louis-Bar"[Text Word] OR "Louis Bar"[Text Word])) AND (neuropsychometric[Text Word] OR IQ[Text Word] OR "cognitive function"[Text Word] OR intell*[Text Word])
- (("Ataxia-telangiectasia"[Text Word] OR "Ataxia-telangectasia"[Text Word] OR "Ataxia telangiectasia"[Text Word] OR "Ataxia telangectasia"[Text Word] OR "Louis-Bar"[Text Word] OR "Louis Bar"[Text Word])) AND ("mental state"[Text Word] OR mood[Text Word] OR "anxiety"[Text Word] OR depression[Text Word])
- (("Ataxia-telangiectasia"[Text Word] OR "Ataxia-telangectasia"[Text Word] OR "Ataxia telangiectasia"[Text Word] OR "Ataxia telangectasia"[Text Word] OR "Louis-Bar"[Text Word] OR "Louis Bar"[Text Word])) AND ("quality of life"[Text Word] OR lifestyle[Text Word])

2. Ovid SP (MEDLINE) 1946- present HUMANS only and 3.)OVID EMBASE 1980 – present (HUMAN ONLY)

a) Exp Ataxia-telangiectasia/ OR ataxia-telangiectasia.mp. OR ataxia-telangectasia.mp. OR Louis-Bar.mp.

b) (natural history or pattern*).mp.

c) exp Diagnosis/ OR (diagnosis OR presentation).mp.

d) exp alpha-fetoprotein/ OR (alpha-fetoprotein OR age OR delay OR AFP).mp.

e) exp lung disease/ OR (lung disease OR Respirat* OR sinopulmonary disease OR Bronchi* OR interstitial lung disease OR ILD).mp.

f) (Neurol* OR Neuroimag* OR Neurodisabil*OR nerv* system OR oculomotor apraxia OR cerebell* OR ataxia OR dysarthri* OR movement disorder OR CT OR computed tomography OR MRI OR magnetic resonance imaging OR bradykinesia OR dystonia OR myoclonus OR choreoathetosis OR Neurodegen* OR tremor OR PET OR positron emission tomography).mp.

g) exp Growth/ OR exp Nutrition/ OR (Growth OR nutrition OR malnutrition OR aspiration OR dysphagia OR PEG OR gastro* OR gastrostomy OR percutaneous endoscopic gastrostomy).mp.

h) exp Malignancy/ OR exp cancer/ OR (malignancy OR cancer OR leukaemia OR leukemia OR tumour OR tumor OR carcinoma OR neoplasm).mp.

i) Exp ataxia-telangiectasia mutated/ OR exp ataxia-telangectasia mutated OR (ataxia-telangiectasia mutated OR ataxia-telangectasia mutated OR Genot* OR phenot* OR genet* OR ATM OR variant).mp.

j) exp infection/ OR (infection OR Immuno* OR immune system OR exp lymph*).mp.

k) exp endocrin*/ OR exp diabetes/ OR (endocrine* OR hormone OR diabetes OR T1DM OR T2DM OR insulin resistance OR vit* D OR vit* E).mp.

l) (skeletal abnormalit* OR scoliosis OR foot deformit* OR fracture OR bone).mp.

m) exp dermatology/ OR (dermatology OR skin OR hyperpigment* OR cafe au lait OR hypopigment* OR telangiectasia OR telangiectasia).mp.

n) exp death/ OR (death OR mortality OR survival).mp.

o) exp fertility/ OR exp pregnancy/ OR (fertility OR pregnancy OR childbirth).mp.

p) (neuropsychometric OR IQ OR cognitive function OR intell*).mp.

q) (mental state OR mood OR anxiety OR depression).mp.

r) Exp quality of life/ OR (quality of life OR lifestyle).mp.

All searches in Ovid carried out with keywords. Then all searches combined with search 2a with ‘AND’.

4 Web of Science core collection

a) TS=("Ataxia-telangiectasia" OR "Ataxia-telangectasia" OR "Ataxia telangiectasia" OR "Ataxia telangectasia" OR “Louis-Bar syndrome” OR “Louis Bar syndrome”) AND TS=(“natural history” OR pattern)

b) TS=("Ataxia-telangiectasia" OR "Ataxia-telangectasia" OR "Ataxia telangiectasia" OR "Ataxia telangectasia" OR “Louis-Bar syndrome” OR “Louis Bar syndrome”) AND TS= (diagnosis OR presentation)

c) TS=("Ataxia-telangiectasia" OR "Ataxia-telangectasia" OR "Ataxia telangiectasia" OR "Ataxia telangectasia" OR “Louis-Bar syndrome” OR “Louis Bar syndrome”) AND TS=(AFP OR “alpha-fetoprotein” OR “alpha fetoprotein” OR age OR delay)

d) TS=("Ataxia-telangiectasia" OR "Ataxia-telangectasia" OR "Ataxia telangiectasia" OR "Ataxia telangectasia" OR “Louis-Bar syndrome” OR “Louis Bar syndrome”) AND TS=(Respirat* OR sinopulmonary disease OR Bronchi* OR interstitial lung disease OR ILD OR lung disease)

e) TS=("Ataxia-telangiectasia" OR "Ataxia-telangectasia" OR "Ataxia telangiectasia" OR "Ataxia telangectasia" OR “Louis-Bar syndrome” OR “Louis Bar syndrome”) AND TS=(Neurol* OR Neuroimag* OR Neurodisabil* OR "nerv* system" OR “oculomotor apraxia” OR cerebell* OR “ataxia” OR dysarthri* OR "movement disorder" OR CT OR “computed tomography" OR MRI OR "magnetic resonance imaging" OR bradykinesia OR dystonia OR myoclonus OR choreoathetosis OR Neurodegen* OR tremor OR PET OR “positron emission tomography”)

f) TS=("Ataxia-telangiectasia" OR "Ataxia-telangectasia" OR "Ataxia telangiectasia" OR "Ataxia telangectasia" OR “Louis-Bar syndrome” OR “Louis Bar syndrome”) AND TS=(Growth OR Nutrition OR malnutrition OR aspiration OR dysphagia OR PEG OR gastro* OR gastrostomy OR ”percutaneous endoscopic gastrostomy”)

g) TS=("Ataxia-telangiectasia" OR "Ataxia-telangectasia" OR "Ataxia telangiectasia" OR "Ataxia telangectasia" OR “Louis-Bar syndrome” OR “Louis Bar syndrome”) AND TS=(Malignancy OR cancer OR leukaemia OR leukemia OR tumour OR tumor OR carcinoma OR neoplasm)

h) TS=("Ataxia-telangiectasia" OR "Ataxia-telangectasia" OR "Ataxia telangiectasia" OR "Ataxia telangectasia" OR “Louis-Bar syndrome” OR “Louis Bar syndrome”) AND TS=(Genot* OR phenot* OR genet* OR ATM OR “Ataxia-telangiectasia mutated " OR "Ataxia-telangectasia mutated " OR "Ataxia telangiectasia mutated " OR "Ataxia telangiectasia mutated " OR variant)

i) TS=("Ataxia-telangiectasia" OR "Ataxia-telangectasia" OR "Ataxia telangiectasia" OR "Ataxia telangectasia" OR “Louis-Bar syndrome” OR “Louis Bar syndrome”) AND TS=(Immuno* OR immune system OR infection OR lymph*)

j) TS=("Ataxia-telangiectasia" OR "Ataxia-telangectasia" OR "Ataxia telangiectasia" OR "Ataxia telangectasia" OR “Louis-Bar syndrome” OR “Louis Bar syndrome”) AND TS=(endocrin* OR hormone OR diabetes OR T1DM OR T2DM OR “insulin resistance” OR “vit* D” OR “vit* E”)

k) TS=("Ataxia-telangiectasia" OR "Ataxia-telangectasia" OR "Ataxia telangiectasia" OR "Ataxia telangectasia" OR “Louis-Bar syndrome” OR “Louis Bar syndrome”) AND TS=(“skeletal abnormalit*” OR scoliosis OR “foot deformit*” OR fracture OR bone)

l) TS=("Ataxia-telangiectasia" OR "Ataxia-telangectasia" OR "Ataxia telangiectasia" OR "Ataxia telangectasia" OR “Louis-Bar syndrome” OR “Louis Bar syndrome”) AND TS=(dermatology OR skin OR hyperpigment* OR “café au lait” OR hypopigment* OR telangiectasia OR telangectasia)

m) TS=("Ataxia-telangiectasia" OR "Ataxia-telangectasia" OR "Ataxia telangiectasia" OR "Ataxia telangectasia" OR “Louis-Bar syndrome” OR “Louis Bar syndrome”) AND TS= (death OR mortality OR survival)

n) TS=("Ataxia-telangiectasia" OR "Ataxia-telangectasia" OR "Ataxia telangiectasia" OR "Ataxia telangectasia" OR “Louis-Bar syndrome” OR “Louis Bar syndrome”) AND TS=(fertility OR pregnancy OR childbirth)

o) TS=("Ataxia-telangiectasia" OR "Ataxia-telangectasia" OR "Ataxia telangiectasia" OR "Ataxia telangectasia" OR “Louis-Bar syndrome” OR “Louis Bar syndrome”) AND TS=(neuropsychometric OR IQ OR “cognitive function” OR intell*)

p) TS=("Ataxia-telangiectasia" OR "Ataxia-telangectasia" OR "Ataxia telangiectasia" OR "Ataxia telangectasia" OR “Louis-Bar syndrome” OR “Louis Bar syndrome”) AND TS=(“mental state” OR mood OR anxiety OR depression)

q) TS=("Ataxia-telangiectasia" OR "Ataxia-telangectasia" OR "Ataxia telangiectasia" OR "Ataxia telangectasia" OR “Louis-Bar syndrome” OR “Louis Bar syndrome”) AND TS=(“quality of life” OR lifestyle)

5. <u>Scopus</u>

Only the following categories were searched: medicine, ‘biochemistry, genetics and molecular biology’, ‘immunology and microbiology’, neuroscience, ‘pharmacology, toxicology and pharmaceutics’, health professions’

a) TITLE-ABS-KEY (*"Ataxia-telang*ectasia"* OR *"Ataxia telang*ectasia"* OR *"Louis-Bar syndrome"* OR *"Louis Bar syndrome"*) AND TITLE-ABS-KEY (*"natural history"* OR *pattern*)

b) TITLE-ABS-KEY (*"Ataxia-telang*ectasia"* OR *"Ataxia telang*ectasia"* OR *"Louis-Bar syndrome"* OR *"Louis Bar syndrome"*) AND TITLE-ABS-KEY (*diagnosis* OR *presentation*)

c) TITLE-ABS-KEY (*"Ataxia-telang*ectasia"* OR *"Ataxia telang*ectasia"* OR *"Louis-Bar syndrome"* OR *"Louis Bar syndrome"*) AND TITLE-ABS-KEY (*AFP* OR *"alpha-fetoprotein"* OR *"alpha fetoprotein"* OR *age* OR *delay*)

d) TITLE-ABS-KEY ("Ataxia-telang*ectasia" OR "Ataxia telang*ectasia" OR "Louis-Bar syndrome" OR "Louis Bar syndrome") AND TITLE-ABS-KEY (Respirat* OR "sinopulmonary disease" OR Bronchi* OR "interstitial lung disease" OR ILD OR "lung disease")

e) TITLE-ABS-KEY (*"Ataxia-telang*ectasia"* OR *"Ataxia telang*ectasia"* OR *"Louis-Bar syndrome"* OR *"Louis Bar syndrome"*) AND TITLE-ABS-KEY (Neurol* OR Neuroimag* OR Neurodisabil* OR “nerv* system” OR “oculomotor apraxia” OR cerebell* OR ataxia OR dysarthri* OR “movement disorder” OR CT OR “computed tomography” OR MRI OR “magnetic resonance imaging” OR bradykinesia OR dystonia OR myoclonus OR choreoathetosis OR Neurodegen* OR tremor OR PET OR “positron emission tomography”)

f) TITLE-ABS-KEY (*"Ataxia-telang*ectasia"* OR *"Ataxia telang*ectasia"* OR *"Louis-Bar syndrome"* OR *"Louis Bar syndrome"*) AND TITLE-ABS-KEY (*growth* OR *nutrition* OR *malnutrition* OR *aspiration* OR *dysphagia* OR *peg* OR *gastro** OR *gastrostomy* OR *"percutaneous endoscopic gastrostomy"*)

g) TITLE-ABS-KEY ("Ataxia-telang*ectasia" OR "Ataxia telang*ectasia" OR "Louis-Bar syndrome" OR "Louis Bar syndrome") AND TITLE-ABS-KEY (Malignancy OR cancer OR leukaemia OR leukemia OR tumour OR tumor OR carcinoma OR neoplasm)

h) TITLE-ABS-KEY (*"Ataxia-telang*ectasia"* OR *"Ataxia telang*ectasia"* OR *"Louis-Bar syndrome"* OR *"Louis Bar syndrome"*) AND TITLE-ABS-KEY (Genot* OR phenot* OR genet* OR ATM OR “Ataxia-telangiectasia mutated” OR “Ataxia-telangectasia mutated” OR “Ataxia telangiectasia mutated” OR “Ataxia telangectasia mutated” OR variant)

i) TITLE-ABS-KEY (*"Ataxia-telang*ectasia"* OR *"Ataxia telang*ectasia"* OR *"Louis-Bar syndrome"* OR *"Louis Bar syndrome"*) AND TITLE-ABS-KEY (*immuno** OR *"immune system"* OR *infection* OR *lymph**)

j) TITLE-ABS-KEY ("Ataxia-telang*ectasia" OR "Ataxia telang*ectasia" OR "Louis-Bar syndrome" OR "Louis Bar syndrome") AND TITLE-ABS-KEY (endocrin* OR hormone OR diabetes OR T1DM OR T2DM OR "insulin resistance" OR "vit* D" OR "vit* E")

k) TITLE-ABS-KEY (*"Ataxia-telang*ectasia"* OR *"Ataxia telang*ectasia"* OR *"Louis-Bar syndrome"* OR *"Louis Bar syndrome"*) AND TITLE-ABS-KEY (*"skeletal abnormalit*"* OR *scoliosis* OR *"foot deformit*"* OR *fracture* OR *bone*)

l) TITLE-ABS-KEY ("Ataxia-telang*ectasia" OR "Ataxia telang*ectasia" OR "Louis-Bar syndrome" OR "Louis Bar syndrome") AND TITLE-ABS-KEY (dermatology OR skin OR hyperpigment* OR "café au lait" OR hypopigment* OR telangiectasia OR telangectasia)

m) TITLE-ABS-KEY ("Ataxia-telang*ectasia" OR "Ataxia telang*ectasia" OR "Louis-Bar syndrome" OR "Louis Bar syndrome") AND TITLE-ABS-KEY (death OR mortality OR survival)

n) TITLE-ABS-KEY (*"Ataxia-telang*ectasia"* OR *"Ataxia telang*ectasia"* OR *"Louis-Bar syndrome"* OR *"Louis Bar syndrome"*) AND TITLE-ABS-KEY (*fertility* OR *pregnancy* OR *childbirth*)

o) TITLE-ABS-KEY (*"Ataxia-telang*ectasia"* OR *"Ataxia telang*ectasia"* OR *"Louis-Bar syndrome"* OR *"Louis Bar syndrome"*) AND TITLE-ABS-KEY (*neuropsychometric* OR *iq* OR *"cognitive function"* OR *intell**)

p) TITLE-ABS-KEY (*"Ataxia-telang*ectasia"* OR *"Ataxia telang*ectasia"* OR *"Louis-Bar syndrome"* OR *"Louis Bar syndrome"*) AND TITLE-ABS-KEY (“mental state” OR mood OR anxiety OR depression)

q) TITLE-ABS-KEY (*"Ataxia-telang*ectasia"* OR *"Ataxia telang*ectasia"* OR *"Louis-Bar syndrome"* OR *"Louis Bar syndrome"*) AND TITLE-ABS-KEY (“quality of life” OR lifestyle)

Prospero and Cochrane Library - 28/3/18 – no systematic reviews completed or registered
