# Supplementary material for: The natural history of ataxia-telangiectasia (A-T): A systematic review
Source: PLoS One. 2022 Mar 15;17(3):e0264177. doi: 10.1371/journal.pone.0264177 (PMC9049793; doi:10.1371/journal.pone.0264177)
Supplement: S1 File — Table S1 Outcome definitions and, Table S2 Study type definitions and, Table S3 Reported antenatal problems and, Table S4 Reported birth weight and gestation and, Table S5 Reported postnatal problems and, Table S6 Detailed cause of death and, Table S7 Quality assessment Case-control studies and, Table S8 Quality assessment Cohort studies. (PDF) [file pone.0264177.s003.pdf]

Supplementary tables

Table S1 – Outcome definitions

Outcome	Definition
**Delay in diagnosis**	Time between presentation to a clinician and the time of diagnosis.
**Presenting sign or symptom**	The first sign or symptom that a parent, child, or adult notices that leads to a diagnosis of A-T
**Gait ataxia**	'Cerebellar gait ataxia', 'gait ataxia', 'unsteady gait' with a diagnosis of A-T or 'staggering gait' with a diagnosis of A-T.
**Dysarthria**	Author reported dysarthria, or slurred speech with a diagnosis of A-T
**Recurrent infection**	Any infection reported as recurrent by the authors of the study, or when 3 or more episodes of the same infection have been reported in 1 year.
**Impaired cognitive function**	Documented as impaired by authors of the paper, or IQ documented at 80 or less
**Immunoglobulins**	A result was regarded as low, normal or high based on the normal ranges documented within each study. Alternatively they were included in the analysis if the study documented that specific immunoglobulin results was low, normal or high. The normal ranges varied slightly. Units of measure were converted if necessary.

Table S2 – Study type definitions

Study type	Definition
**Case report**	Single case of A-T described in the study.
**Case series**	More than 1 case described in detail, generally as individual cases in the same study.
**Cohort study**	A longitudinal study of a group of people with A-T where individual cases are not described in detail.
**Cross-sectional study**	Results were collected at one moment in time and the cases were not described in detail, or were grouped together for analysis
**Interventional study**	Participants received an intervention.
**Case-control study**	A group of people with A-T-were compared to a control group, a group of people without A-T.
**Qualitative study**	A descriptive study in which non-numerical data was collected.
**Prevalence study**	A whole population with a particular feature was studied at a particular point in time.
Many articles had multiple study types within them. In this case the most appropriate type of study compared to the definitions above was selected. The same data was collected for all study types.	

Table S3 – Reported antenatal problems

Antenatal problem	Number of studies (references)	Number with antenatal problem
**Total number**	**65**	**180**
**Total with antenatal problem**	**12**	**21**
**Undefined antenatal problems**	1	4
**Breech delivery**	2	2
**Antepartum bleeding**	4	4
**Low birth weight**	1	1
**Forceps delivery**	1	2
**Signs of fetal hypoxia**	1	1
**Maternal pyelitis**	1	1
**Premature rupture of membranes (PROM)**	1	1
**Intrauterine growth restriction (IUGR)**	1	1
**Seizures**	1	1
**Rubella contact**	1	1
**Hyperemesis**	1	1
**Single nuchal cord**	1	1
**No antenatal problems**	**53**	**263**

Table S4 – Reported birth weight and gestation

Birth weight	n	Range (kg)	IQR (kg)
**Overall**	41	1.30 – 4.08	2.38 – 3.30
**Term infants**	21	1.9 – 4.08	2.25 – 3.25
**Unknown gestation**	18	1.3 – 3.9	2.6 – 3.4

Table S5 – Reported postnatal problems

Postnatal concern	Number of studies (references)	N	Age
**Number of cases reported**	**35**	46	N/A
**Postnatal concerns**	**12**	23	N/A
**Abnormal newborn screening**	5	15	N/A
**Jaundice**	4	4	32 weeks, 39 weeks x 2 unknown
**Neonatal asphyxia**	2	2	Unknown
**Neonatal resuscitation required**	1	1	Unknown
**Abnormal sutures**	1	1	>37 weeks
**Poor feeding**	1	1	32 weeks
**Oxygen in neonatal period**	2	2	Unknown
**No postnatal concerns**	**23**	23	11 x >37 weeks, 11 x unknown

S6 – Detailed cause of death

Cause of death	Number of studies (references)	All cases		Variant cases		Classical cases	
		Number of cases	Age of death (months)	Number of cases	Age of death (months)	Number of cases	Age of death (months)
**Neurological**							
**Acute increase in ICP**	1 [278]	2	n = 2<br>336.0, 336.0	0	Not applicable	2	n=2<br>336.0, 336.0
**Unspecified disease**	1 [874]	10	Not reported	0	Not applicable	10	Not reported
**Complication of raised ICP**	1 [668]	5	Not reported	0	Not applicable	5	Not reported
**Encephalopathy**	1 [160]	1	n=1<br>32.0	0	Not applicable	1	Not reported
**Widespread thrombotic microangiopath y**	1 [693]	1	n = 1<br>384.0	0	Not applicable	1	n=1<br>384.0
**NEUROLOGICAL TOTAL**	N/A	18	n = 3<br>32.0, 336.0, 336.0	0	Not applicable	18	n=3<br>32.0, 336.0, 336.0
**A-T related**							
**A-T (not specified)**	1 [951]	1	n=1<br>192.0	0	Not applicable	1	n=1<br>96.0

Cause of death	Number of studies (references)	All cases		Variant cases		Classical cases	
		Number of cases	Age of death (months)	Number of cases	Age of death (months)	Number of cases	Age of death (months)
**Complications of A-T**	3 [818, 866, 951]	5	n=3 64.8, 134.4, 288.0	0	Not applicable	5	n=3 64.8, 134.4, 288.0
**General deterioration**	1 [467]	1	n=1 108.0	0	Not applicable	1	n=1 120.0
**Disease Progression**	1 [474]	1	n=1 84.0	0	Not applicable	1	n=1 126.0
**A-T TOTAL**	N/A	8	n=6 median (range, IQR) 121.2 (64.8-288.0, 90.0-177.6)	0	Not applicable	8	n=6 median (range, IQR) 121.2 (64.8-288.0; 90.0-177.6)
**Haemorrhage/Bleeding**							
**Unspecified Bleeding**	1 [881]	2	Not reported	0	Not applicable	2	Not reported
**Bladder haemorrhage**	2 [52, 448]	2	n=1 360.0	0	Not applicable	2	n=1 132.0
**Cerebral haemorrhage**	3 [347, 789, 949]	3	n=3 36.0, 312.0, 408.0	0	Not applicable	2	n=3 36.0, 312.0, 408.0
**GI haemorrhage**	1 [575]	1	n=1 180.0	0	Not applicable	1	n=1 144.0

Cause of death	Number of studies (references)	All cases		Variant cases		Classical cases	
		Number of cases	Age of death (months)	Number of cases	Age of death (months)	Number of cases	Age of death (months)
**Haemoptysis**	1 [116]	1	n=1 132.0	0	Not applicable	1	n=1 180.0
**Urinary tract haemorrhage**	2 [24, 49]	2	n=2 264.0, 264.0	0	Not applicable	2	n=2 264.0, 264.0
**BLEEDING TOTAL**	N/A	11	n=8 median (range, IQR) 264.0 (36.0-408.0, 168.0-324.0)	0	Not applicable	11	n=8 median (range, IQR) 264.0 (36.0-408.0, 168.0-324.0)
			**Malignancy**				
**ALL**	10 [24, 35, 36, 52, 87, 229, 339, 376, 532, 626]	15	n=12 median (range, IQR) 134.0 (48.0-612.0, 69.0-221.5)	3	n=3 156.0, 612.0, 612.0	12	n=9 median (range, IQR) 72.0 (48.0-238.0, 60.0-188.0)
**AML**	1 [264]	1	n=1 120.0	0	Not applicable	1	n=1 120.0
**B-cell lymphoma**	4 [24, 177, 614, 789]	9	n=9 median (range, IQR) 132.0 (120.0-480.0, 120.0-204.0)	0	Not applicable	9	n=9 median (range, IQR) 132.0 (120.0-480.0, 120.0-204.0)
**Breast cancer**	3 [24, 28, 36]	4	n=4 median (range) 396.0 (324.0-564.0)	2	n=2 396.0, 564.0	2	n=2 324.0, 396.0

Cause of death	Number of studies (references)	All cases		Variant cases		Classical cases	
		Number of cases	Age of death (months)	Number of cases	Age of death (months)	Number of cases	Age of death (months)
**Burkitt's lymphoma**	4 [87, 626, 696, 758]	10	n=7 median (range, IQR) 196.0 (59.0-230.0, 114.0-220.5)	0	Not applicable	10	n=7 median (range, IQR) 196.0 (59.0-230.0; 114.0-220.5)
**Cerebellar tumour**	1 [296]	1	n=1 156.0	0	Not applicable	1	n=1 156.0
**CML**	1 [24]	1	n=1 564.0	1	n=1 564.0	0	Not applicable
**Ectopic pituitary tumour**	1 [52]	1	n=1 276.0	1	n=1 276.0	0	Not applicable
**Fetal hepatoblastoma**	1 [869]	1	n=1 168.0	0	Not applicable	1	n=1 288.0
**Gallbladder malignancy**	1 [72]	1	n=1 840.0	0	Not applicable	1	n=1 840.0
**Gastric adenocarcinoma**	1 [91]	1	n=1 312.0	0	Not applicable	1	n=1 372.0
**Gastric cancer**	2 [185, 782]	2	n=1 168.0	0	Not applicable	2	n=1 372.0

Cause of death	Number of studies (references)	All cases		Variant cases		Classical cases	
		Number of cases	Age of death (months)	Number of cases	Age of death (months)	Number of cases	Age of death (months)
**HD lymphocyte depleted**	1 [626]	2	n=2 152.0, 249.0	0	Not applicable	2	n=2 152.0, 249.0
**HD with nodular sclerosis**	1 [626]	2	n=2 85.0, 215.0	0	Not applicable	2	n=2 85.0, 215.0
**Hepatocellular carcinoma**	7 [24, 49, 52, 225, 294, 789, 811]	7	n=7 median (range, IQR) 168.0 (120.0-444.0, 120.0-420.0)	1	n=1 396.0	5	n=5 median (range, IQR) 144.0 (120.0-444.0; 120.0-375.0)
**Hepatoma**	2 [312, 810]	2	n=1 204.0	0	Not applicable	2	n=1 204.0
**HL**	9 [24, 52, 87, 133, 204, 355, 614, 696, 819]	12	n=8 median (range, IQR) 120.0 (60.0-312.0, 99.0-156.0)	0	Not applicable	12	n=8 median (range, IQR) 120.0 (60.0-312.0, 99.0-156.0)
**Lymphoblastic lymphoma**	1 [626]	2	n=2 161.0, 258.0	0	Not applicable	2	n=2 161.0, 258.0
**Lymphoblastic lymphosarcoma**	1 [376]	1	Not reported	0	Not applicable	1	Not reported
**Lymphoid malignancy**	1 [626]	24	Not reported	0	Not applicable	24	Not reported

Cause of death	Number of studies (references)	All cases		Variant cases		Classical cases	
		Number of cases	Age of death (months)	Number of cases	Age of death (months)	Number of cases	Age of death (months)
**Lymphoreticular malignancy**	1 [287]	5	n=5 median (range, IQR) 180.0 (108.0-504.0, 144.0-204.0)	0	Not applicable	5	n=5 median (range, IQR) 180.0 (108.0-504.0; 144.0-204.0)
**Lymphosarcoma**	1 [407]	1	n=1 54.0	0	Not applicable	1	n=1 54.0
**Malignant lymphoproliferative disorder**	1 [669]	1	n=1 384.0	0	Not applicable	1	n=1 384.0
**Multiple gliovascular lesions**	1 [347]	1	n=1 456.0	0	Not applicable	1	n=1 456.0
**Neuroectodermal tumour of the brain**	1 [580]	2	n=1 96.0	0	Not applicable	2	n=1 96.0
**NHL**	8 [36, 373, 532, 614, 696, 789, 790, 874, 880]	17	n=4 24.0, 48.0, 72.0, 264.0	0	Not applicable	17	n=4 24.0, 48.0, 72.0, 264.0
**NHL Large cell**	1 [626]	11	n=11 median (range, IQR) 219.0 (89.0-320.0, 153.0-287.0)	0	Not applicable	11	n=11 median (range, IQR) 219.0 (89.0-320.0, 153.0-287.0)

Cause of death	Number of studies (references)	All cases		Variant cases		Classical cases	
		Number of cases	Age of death (months)	Number of cases	Age of death (months)	Number of cases	Age of death (months)
**Ovarian dysgerminoma**	1 [312]	1	Not reported	0	Not applicable	1	Not reported
**Pancreatic adenocarcinoma**	1 [33]	1	n=1 600.0	1	n=1 600.0	0	Not applicable
**Pancreatic cancer**	1 [35]	1	n=1 576.0	1	n=1 576.0	0	Not applicable
**Prostate cancer**	2 [24, 498]	2	n=2 636.0, 648.0	0	Not applicable	2	n=2 636.0, 648.0
**Refractory lymphoma**	1 [794]	1	Not reported	0	Not applicable	1	Not reported
**Sigmoid adenocarcinoma**	1 [764]	1	n=1 96.0	0	Not applicable	1	n=1 198.0
**Small cell lymphosarcoma**	1 [341]	1	Not reported	0	Not applicable	1	Not reported
**T-ALL**	3 [124, 614, 789]}	3	n=3 24.0 108.0, 144.0	0	Not applicable	3	n=2 24.0, 108.0, 144.0
**T-cell lymphoma**	3 [24, 93, 696]	4	n=1 108.0	0	Not applicable	4	n=1 108.0
**T-PLL**	1	1	n=1	0	Not applicable	1	n=1

Cause of death	Number of studies (references)	All cases		Variant cases		Classical cases	
		Number of cases	Age of death (months)	Number of cases	Age of death (months)	Number of cases	Age of death (months)
	[79]		228.0				228.0
**Undefined pancreatic cancer**	1 [24]	1	n=1 576.0	1	n=1 576.0	0	Not applicable
**Unspecified Leukaemia**	7 [36, 72, 76, 108, 142, 495, 696]	7	n=3 72.0, 123.0, 192.0	2	n=2 564.0, 912.0	5	n=3 72.0, 123.0, 192.0
**Unspecified lymphoma**	12 [24, 90, 142, 250, 264, 294, 378, 382, 510, 810, 853, 854]	18	n=12 median (range, IQR) 120.0 (72.0-288.0, 117.0-162.0)	0	Not applicable	18	n=12 median (range, IQR) 120.0 (72.0-288.0, 117.0-162.0)
**Unspecified malignancy**	24 [36, 43, 52, 61, 66, 76, 80, 201, 208, 211, 290, 337, 346, 370, 449, 490, 576, 716, 793, 810, 818, 866, 881, 885]}	128	n=11 median (range, IQR) 264.0 (45.0-612.0, 103.0-360.0)	4	n=2 110.0, 612.0	124	n=9 median (range, IQR) 264.0 (45.0-408.0; 96.0-312.0)
**Widespread metastatic disease**	1 [569]	1	n=1 48.0	0	Not applicable	1	n=1 48.0
**CANCER TOTAL**	N/A	308	n=125 median (range, IQR) 167.0 (24.0-912.0, 108.0-294.0)	14	n=14 median (range, IQR) 564.0 (110-612.0, 396.0-594.0)	266	n=101 median (range, IQR) 156.0 (24.0-840.0, 108.0-253.3)
**Cardiac**							

Cause of death	Number of studies (references)	All cases		Variant cases		Classical cases	
		Number of cases	Age of death (months)	Number of cases	Age of death (months)	Number of cases	Age of death (months)
**Congestive heart failure**	1 [696]	2	Not reported	0	Not applicable	2	Not reported
**Pulmonary hypertension**	1 [24]	1	n=1 48.0	0	Not applicable	1	n=1 48.0
**Ventricular fibrillation**	1 [354]	1	n=1 93.0	0	Not applicable	1	n=1 93.0
**CARDIAC TOTAL**	N/A	4	n=2 48.0, 93.0	0	Not applicable	4	n=2 48.0, 93.0
**Gastrointestinal**							
**Decompensate d cirrhosis**	1 [949]	1	n=1 312.0	0	Not applicable	1	n=1 312.0
**Hepatic failure**	2 [160, 211, 457]	3	n=2 32.0, 144.0	0	Not applicable	3	n=2 32.0, 144.0
**GI TOTAL**	N/A	3	n=3 32.0, 144.0, 312.0	0	Not applicable	3	n=3 32.0, 144.0, 312.0
**Haematology (non-malignant)**							
**Haemophagocy tic syndrome**	1 [532]	1	Not reported	0	Not applicable	1	Not reported
**HAEMATOLOGI CAL TOTAL**	N/A	1	Not reported	0	Not applicable	1	Not reported

Cause of death	Number of studies (references)	All cases		Variant cases		Classical cases	
		Number of cases	Age of death (months)	Number of cases	Age of death (months)	Number of cases	Age of death (months)
**Infection (excluding pneumonia)**							
**Disseminated HSV**	1 [319]	1	n=1 84.0	0	Not applicable	1	n=1 84.0
**Measles**	1 [407]	1	n=1 42.0	0	Not applicable	1	n=1 42.0
**Meningitis**	1 [341]	1	Not reported	0	Not applicable	1	Not reported
**Pulmonary infection**	17 [78, 87, 114, 132, 139, 177, 201, 256, 345, 352, 432, 458, 532, 541, 614, 880, 953]	67	n=17 median (range, IQR) 132.0 (108.0-240.0, 120.0-192.0)	0	Not applicable	67	n=11 median (range, IQR) 132.0 (108.0-240.0; 120.0-192.0)
**Systemic candidiasis**	1 [76]	1	Not reported	0	Not applicable	1	Not reported
**TB**	1 [407]	1	n=1 132.0	0	Not applicable	1	n=1 132.0
**Unspecified infection**	13 [76, 158, 317, 337, 346, 376, 387, 564, 624, 769, 790, 854, 881]	59	n=5 median (range, IQR) 153.6 (72.0-240.0, 82.6-192.0)	0	Not applicable	59	n=5 median (range, IQR) 153.6 (72.0-240.0; 82.6-192.0)
**INFECTION TOTAL**	N/A	129	n=19	0	Not applicable	129	n=19

Cause of death	Number of studies (references)	All cases		Variant cases		Classical cases	
		Number of cases	Age of death (months)	Number of cases	Age of death (months)	Number of cases	Age of death (months)
**(excluding pneumonia)**			median (range, IQR) 132.0 (42.0-240.0, 114.0-186.0)				median (range, IQR) 132.0 (42.0-240.0; 114.0-186.0)
**Malignancy related complications**							
**Consequences of lymphoproliferative disorder**	2 [452, 678]	2	Not reported	0	Not applicable	2	Not reported
**Infectious complications during chemotherapy**	1 [855]	1	Not reported	0	Not applicable	1	Not reported
**Pre-leukaemic syndrome**	1 [824]	1	Not reported	0	Not applicable	1	Not reported
**Consequences of radiotherapy**	1 [578]	1	Not reported	0	Not applicable	1	Not reported
**Toxicity**	3 [337, 614, 789, 885]}	14	n=3 120.0, 178.8, 300.0	0	Not applicable	13	n=2 120.0, 178.8, 300.0
**Complications of GI cancer**	1 [190]	1	n=1 288.0	0	Not applicable	1	n=1 288.0
**MALIG RELATED TOTAL**	N/A	19	n=4 120.0, 178.8, 288.0, 300.0	0	Not applicable	19	n=4 120.0, 178.8, 288.0, 300.0

Cause of death	Number of studies (references)	All cases		Variant cases		Classical cases	
		Number of cases	Age of death (months)	Number of cases	Age of death (months)	Number of cases	Age of death (months)
**Multi-organ failure**							
**MULTI-ORGAN FAILURE**	5 [166, 220, 397, 815, 821]	7	n=4 90.0, 96.0 132.0, 144.0	0	Not applicable	7	n=4 90.0, 96.0 132.0, 144.0
**Nutritional**							
**Cachexia**	2 [36, 57]	2	n=2 468.0, 720.0	1	n=1 720.0	1	n=1 468.0
**Severe malnutrition**	1 [764]	1	Not reported	0	Not applicable	1	Not reported
**Severe prostration**	1 [276]	1	n=1 177.0	0	Not applicable	1	n=1 177.0
**Wasting**	1 [391]	1	n=1 240.0	0	Not applicable	1	n=1 240.0
**NUTRITION TOTAL**	N/A	5	n=4 median (range) 354.0 (177.0-720.0)	1	n=1 720	4	n=3 354.0, 177.0, 720.0
**Other causes**							
**EBV lymphoprolifera tion**	1 [789]	1	n=1 204.0	0	Not applicable	1	n=1 204.0

Cause of death	Number of studies (references)	All cases		Variant cases		Classical cases	
		Number of cases	Age of death (months)	Number of cases	Age of death (months)	Number of cases	Age of death (months)
**Lymphadogranelematosis**	1 [76]	1	n=1 108.0	0	Not applicable	1	n=1 108.0
**Haemophagocytosis**	1 [78]	1	Not reported	0	Not applicable	1	Not reported
**Post-op complications**	1 [483]	1	n=1 108.0	0	Not applicable	1	n=1 108.0
**Bowel obstruction**	1 [789]	1	n=1 300.0	0	Not applicable	1	n=1 300.0
**OTHER TOTAL**	N/A	5	n=4 108.0, 108.0, 204.0, 300.0	0	Not applicable	5	n=4 108.0, 108.0, 204.0, 300.0
**Pneumonia**							
**Aspiration pneumonia**	2 [278, 696]	3	n=1 300.0	0	Not applicable	3	n=1 300.0
**Other Pneumonia**	37 [49, 76, 99, 108, 121, 128, 134, 142, 221, 242, 278, 294, 296, 341, 347, 358, 382, 385, 431, 438, 442, 445, 458, 513, 572, 648, 657, 693, 696, 719, 771, 782, 789, 821, 853, 951]}	62	n=33 median (range, IQR) 168.0 (15.0-564.0, 120.0-300.0)	1	n=1 396.0	50	n=34 median (range, IQR) 162.0 (15.0-564.0, 120.0-300.0)

Cause of death	Number of studies (references)	All cases		Variant cases		Classical cases	
		Number of cases	Age of death (months)	Number of cases	Age of death (months)	Number of cases	Age of death (months)
**PNEUMONIA TOTAL**	N/A	54	n=31<br>median (range, IQR)<br>177.0 (15.0-564.0, 123.0-300.0)	1	396.0	53	n=30<br>median (range, IQR)<br>168.0 (15.0-564.0; 120.0-300.0)
				**Renal**			
**Acute renal failure**	1<br>[842]	1	Not reported	0	Not applicable	1	Not reported
**Renal failure**	1<br>[818]	1	n=1<br>97.2	0	Not applicable	1	n=1<br>97.2
**RENAL TOTAL**	N/A	2	n=1<br>97.2	0	Not applicable	2	n=1<br>97.2
				**Respiratory failure**			
**RESPIRATORY FAILURE**	32<br>[24, 36, 49, 52, 128, 131, 134, 139, 142, 204, 211, 221, 225, 240, 294, 302, 305, 347, 358, 464, 495, 498, 513, 572, 614, 693, 696, 717, 730, 774, 789, 834, 853, 864, 950-952]	92	n=49<br>median (range, IQR) 216.0 (108.0-600.0, 144.0-300.0)	0	Not reported	92	n=49<br>median (range, IQR) 216.0 (108.0-600.0, 144.0-300.0)
				**Other respiratory causes**			
**Acute saturation drop**	1	1	n=1	0	Not applicable	1	n=1

Cause of death	Number of studies (references)	All cases		Variant cases		Classical cases	
		Number of cases	Age of death (months)	Number of cases	Age of death (months)	Number of cases	Age of death (months)
	[763]		156.0				156.0
**ARDS**	1 [513]	1	Not reported	0	Not applicable	1	Not reported
**Bronchiectasis**	1 [211]	7	Not reported	0	Not applicable	7	Not reported
**Bronchitis**	1 [242]	1	n=1 168.0	0	Not applicable	1	n=1 168.0
**Chronic lung disease**	2 [87, 614]	5	Not reported	0	Not applicable	5	Not reported
**Chronic pulmonary disease**	1 [75]	1	n=1 84.0	0	Not applicable	1	n=1 84.0
**Interstitial lung disease**	1 [789]	2	n=2 120.0, 216.0	0	Not applicable	2	n=2 120.0, 216.0
**Pleural effusion**	1 [290]	1	n=1 408.0	0	Not applicable	1	n=1 408.0
**Pneumocystis**	1 [818]	1	Not reported	0	Not applicable	1	Not reported
**Pneumonitis**	1 [513]	1	Not reported	0	Not applicable	1	Not reported

Cause of death	Number of studies (references)	All cases		Variant cases		Classical cases	
		Number of cases	Age of death (months)	Number of cases	Age of death (months)	Number of cases	Age of death (months)
**Pneumothorax**	2 [345, 696]	3	n=1<br>132.0	0	Not applicable	3	n=1<br>132.0
**Pulmonary complications**	3 [427, 510, 615]	15	n=1<br>180.0	0	Not applicable	15	n=1<br>180.0
**Pulmonary fibrosis**	1 [310]	1	n=1<br>213.0	0	Not applicable	1	n=1<br>213.0
**Respiratory deterioration**	1 [255]	3	n=3<br>96.0, 96.0, 108.0	0	Not applicable	3	n=3<br>96.0, 96.0, 108.0
**Unspecified respiratory cause**	3 [696, 874, 881]	8	Not reported	0	Not applicable	8	Not reported
**RESPIRATORY OTHER TOTAL**	N/A	51	n=12<br>median (range, IQR)<br>144.0 (84.0-408.0, 105.0-188.3)	0	Not applicable	51	n=12<br>median (range, IQR)<br>144.0 (84.0-408.0, 105.0-188.3)
**Sepsis**							
**SEPSIS**	15 [76, 78, 121, 155, 240, 278, 310, 410, 449, 571, 603, 614, 789, 818, 842]	18	n=8<br>median (range, IQR)<br>208.5 (60.0-312.0, 180.0-237.0)	0	Not applicable	18	n=8<br>median (range, IQR)<br>208.5 (60.0-312.0; 180.0-237.0)
**Unknown cause**							

Cause of death	Number of studies (references)	All cases		Variant cases		Classical cases	
		Number of cases	Age of death (months)	Number of cases	Age of death (months)	Number of cases	Age of death (months)
**UNKNOWN**	86 [24, 36, 61, 62, 70, 75, 76, 78, 88, 90, 127, 130, 139, 154, 156, 162-164, 175, 176, 187, 205, 231, 270, 275, 298, 304, 327, 337, 346, 350, 364, 374, 394, 400, 401, 407-409, 424, 434, 449, 473, 489, 565, 568, 577, 610, 615, 626, 638, 660, 676, 681, 686, 694, 735, 758, 764, 788-792, 794, 822, 825, 833, 844, 858, 865, 866, 882, 884, 888, 890, 954-963]	1021	n=40 median (range, IQR) 192.0 (24.0-612.0, 130.5-294.0)	2	n=2 492.0, 612.0	1019	n=38 median (range, IQR) 186.0 (24.0-552.0; 129.5-279.0)

S7 – Quality assessment Case-control studies

Title	Author	Type of article	Case definition adequate	Representative of the cases	Selection of controls	Definition of controls	Comparability	Ascertainment of exposure	Same method for cases and controls	Non-response rate	Quality
Altered corticomotor-cerebellar integrity in young ataxia telangiectasia patients	I. Sahama et al	Full text	A*	A*	C	A*	A*	A*	A*	A*	7
Antibody deficiency in patients with ataxia telangiectasia is caused by disturbed B- and T-cell homeostasis and reduced immune repertoire diversity	G. J. Driessen et al	Full text	A*	A*	C	A*	A,B **	A*	A*	A*	8
Antibody response to a seven-valent pneumococcal conjugated vaccine in patients with ataxia-telangiectasia	O. Sanal et al	Full text	A*	A*	A*	A*	A,B **	A*	A*	A*	9
Antibody responses in vivo in chromosome instability syndromes with immunodeficiency	C. M. Weemaes et al	Full text	C	B	C	A*	None	A*	A*	A*	4
Ataxia Telangiectasia (AT) as a radiation sensitivity syndrom and limits of radiotherapeutic intervention	F. T. Hoche et al	Full text (conf abstract)	A*	A*	A*	A*	None	A*	A*	A*	7
Audiological findings in children with ataxia-telangiectasia (A-T) syndrome	P. O. Afifi, H. H Elsanadiky	Full text	A*	B	A*	A*	None	A*	A*	A*	6
Autonomic dysfunction in patients with Ataxia-Telangiectasia	L. Tubani et al	Full text	A*	B	A*	A*	A,B **	A*	A*	A*	8
Blood metal levels and related antioxidant enzyme activities in patients with ataxia telangiectasia	S. Squadrone et al	Full text	A*	A*	A*	A*	A,B **	A*	A*	A*	9

Title	Author	Type of article	Case definition adequate	Representative of the cases	Selection of controls	Definition of controls	Comparability	Ascertainment of exposure	Same method for cases and controls	Non-response rate	Quality
Body composition, muscle strength and hormonal status in patients with ataxia telangiectasia: a cohort study	H. Pommerening et al	Full text	A*	A*	A*	A*	A,B **	A*	A*	A*	9
Brain glucose metabolism in adults with ataxia-telangiectasia and their asymptomatic relatives	N. D. Volkow et al	Full text	A*	A*	A*	A*	A,B **	A*	A*	A*	9
Cerebral glutathione is unchanged in children with AT: Preliminary results from the children ataxia teleangiectasia neuro assessment project (CATNAP)	F. Raschke et al	Full text (conf abstract)	B	B	C	A*	None	A*	A*	A*	4
Classical ataxia telangiectasia patients have a congenitally aged immune system with high expression of CD95	E. F. Carney et al	Full text	A*	A*	A*	A*	A*	A*	A*	A*	8
Cognitive phenotype in ataxia-telangiectasia	F. Hoche et al	Full text	A*	A*	A*	A*	A,B **	B	A*	A*	8
Conditioning in identical twins with ataxia-telangiectasia	S. H. Mostofsky et al	Full text	A*	A*	A*	A*	A*	A*	A*	A*	8
Coordination between respiration and deglutition in children with Ataxia-Telangiectasia (A-T)	M. A. Lefton-Greif et al	Full text	A*	A*	C	B	A*	A*	A*	A*	6
Decreased cytoplasmic immunoglobulin A production during Epstein-Barr virus immortalization on lymphocytes from patients with ataxia-telangiectasia	M. Okano et al	Full text	A*	B	C	A*	A,B **	A*	A*	A*	7
Defective pneumococcal antibody response in patients with recurrent respiratory tract infections	B. Erman et al	Full text	A*	A*	B	A*	A*	A*	A*	A*	7
Deficiencies in CD4+ and CD8+ T cell subsets in ataxia telangiectasia	R. R. Schubert et al	Full text	A*	A*	C	A*	A	A*	A*	A*	6

Title	Author	Type of article	Case definition adequate	Representative of the cases	Selection of controls	Definition of controls	Comparability	Ascertainment of exposure	Same method for cases and controls	Non-response rate	Quality
Disorders of B cells and helper T cells in the pathogenesis of the immunoglobulin deficiency of patients with ataxia telangiectasia	T. A. Waldmann et al	Full text	A*	B	C	B	None	A*	B	A*	3
Disorders of Upper Limb Movements in Ataxia-Telangiectasia	A. G. Shaikh et al	Full text	A*	A*	A*	A*	None	A*, C	A*	A*	7
Dyslipidemia and reduced antioxidant vitamins in ataxia telangiectasia patients	I. G. A. Andrade et al	Full text (conf abstract)	B	B	C	A*	None	A*	A*	A*	4
Early diagnosis of ataxia-atelangiectasia using radiosensitivity testing	X. Sun et al	Full text	A*	A*	C	B	None	A	A*	A*	4
Effect of thymic humoral factor on cellular immune factors of normal children and of pediatric patients with ataxia telangiectasia and Down's syndrome	Z. T. Handzel et al	Full text	A*	B	B	A*	A*	A*	A*	A*	6
Elevated serum IL-8 levels in ataxia telangiectasia	S. A. McGrath-Morrow et al	Full text	A*	A*	B	A*	None	A*	A*	A*	6
Epstein-Barr virus (EBV)-specific cell-mediated and humoral immune responses in ataxia-telangectasia patients	G. Masucci et al	Abstract	A*	A*	A*	A*	None	A*	U/K	U/K	5
Epstein-Barr virus antibodies in patients with ataxia-telangiectasia and other immunodeficiency diseases	J. H. Joncas et al	Full text	A*	B	C	A*	None	A*	A*	A*	5
Epstein-Barr virus-related antibody patterns in ataxia-telangiectasia	A. I. Berkel et al	Full text	B	B	A*	A*	A*	A*	A*	A*	6
Evidence that defective gamma interferon production in patients with primary immunodeficiencies is due to intrinsic incompetence of lymphocytes	R. Paganelli et al	Full text	B	B	C	A*	A*	A*	A*	A*	5

Title	Author	Type of article	Case definition adequate	Representative of the cases	Selection of controls	Definition of controls	Comparability	Ascertainment of exposure	Same method for cases and controls	Non-response rate	Quality
Expression of CD40, CD40L and IgM production in patients with ataxiatelangiectasia	C. T. M. Pereira et al	Full text (conf abstract)	B	A*	C	A*	A*	A*	A*	A*	6
Flow cytometry as an important tool in the diagnosis of immunodeficiencies demonstrated in a patient with ataxia-telangiectasia	A. B. Stefano et al	Abstract	A*	A*	A*	A*	None	A*	A*	A*	7
Growth and nutrition in children with ataxia telangiectasia	E. Stewart et al	Full text	A*	A*	C	A*	A*	A*	A*	A*	7
Growth hormone deficiency and immune status in patients with ataxia telangiectasia	S. Vos et al	Full text (conf abstract)	A*	A*	C	A*	A,B **	A*	A*	A*	8
Growth retardation and growth hormone deficiency in patients with Ataxia telangiectasia	S. Voss et al	Abstract	A*	U/K	U/K	A*	U/K	A*	A*	U/K	4
H2AX gene does not have a modifier effect on ataxia-telangiectasia phenotype	L. O. Mesci et al	Full text	A*	A*	C	A*	A,B **	A*	B	A*	7
Health-related quality of life in primary immune deficient patients	H. Mozaffari et al	Full text	B	B	A*	A*	A*	C, D	A*	A*	5
Heterotopic Purkinje cells in ataxia-telangiectasia	A. R. Bottini et al	Full text	A*	A*	B	A*	A,B **	A*	A*	A*	8
Histocompatibility (HLA) factors in ataxia telangiectasia	A. I. Berkel, F. Ersoy	Full text	U/K	U/K	A*	A*	None	A*	A*	U/K	4
Immunoglobulin Content of Intestinal Plasma Cells in Ataxia Telangiectasia	S. Eidelman, S.D. Davis	Full text	B	B	C	A*	A*	A*	A*	A*	5
Immunoglobulin metabolism in ataxia telangiectasia	W. Strober et al	Full text	A*	A*	A*	A*	None	A*	A*	A*	7

Title	Author	Type of article	Case definition adequate	Representative of the cases	Selection of controls	Definition of controls	Comparability	Ascertainment of exposure	Same method for cases and controls	Non-response rate	Quality
Impaired interferon-gamma production in response to live bacteria and Toll-like receptor agonists in patients with ataxia telangiectasia	J. S. Reichenbach et al	Full text	B	B	A*	A*	A*	A*	A*	A*	6
Judgment of duration in individuals with ataxia-telangiectasia	S. H. Mostofsky et al	Full text	A*	A*	C	A*	A*	A*	A*	A*	7
Letter: A.F.P. and ataxia-telangiectasia	M. J. H. Simons et al	Full text	A*	B	C	A*	None	A*	A*	A*	5
Motor pathway degeneration in young ataxia telangiectasia patients: A diffusion tractography study	I. Sahama et al	Full text	A*	A*	C	A*	A*	A*	A*	A*	7
Multimodal electrophysiological assessment of ataxia telangiectasia	M. J. Taylor, W.J. Logan	Full text	B	B	C	A*	A*	A*	A*	A*	5
Novel homozygous ataxia-telangiectasia (A-T) mutated gene mutation identified in a Chinese pedigree with A-T	W.C. Chen et al	Full text	A*	A*	A*	A*	None	A*	A*	A*	7
Nutritional Status, Plasma Levels of Retinol and Beta-Carotene and Oxidative Stress in Ataxia-Telangiectasia Patients	R. Silva et al	Full text (conf abstract)	A*	A*	A*	A*	A*B*	A*	A*	A*	9
Physiological effects of pioglitazone and metformin in patients with ataxia-telangiectasia	L.J. McCreight et al	Abstract and Poster	A*	A*	A*	A*	A*	A*	A*	A*	8
Premature ageing of the immune system underlies immunodeficiency in ataxia telangiectasia	A. R. B. Exley et al	Full text	A*	A*	C	A*	None	A*	A*	A*	6
Proteomic characterization of cerebrospinal fluid from ataxia-telangiectasia (A-T) patients using a LC/MS-based label-free protein quantification technology	M. Dzieciatkowska et al	Full text	A*	B	B	A*	None	A*	A*	A*	5

Title	Author	Type of article	Case definition adequate	Representative of the cases	Selection of controls	Definition of controls	Comparability	Ascertainment of exposure	Same method for cases and controls	Non-response rate	Quality
Proton MR spectroscopic imaging in ataxia-telangiectasia	D. D. Lin et al	Abstract	A*	B	C	A*	A*	A*	A*	A*	6
Proton spectroscopy and imaging at 3T in ataxia-telangiectasia	L. I. Wallis et al	Full text	A*	A*	A*	A*	A*	A*	A*	A*	8
Quantitative evaluation of brain involvement in ataxia telangiectasia by diffusion weighted MR imaging	A. K. Firat et al	Full text	A*	A*	A*	A*	None	A*	A*	A*	7
Risk of Atherosclerosis and Diabetes in Patients with Ataxia Telangiectasia	T. L. Paulino et al	Full text (conf abstract)	A*	A*	C	A*	A,B **	A*	A*	A*	8
Screening test for ataxia telangiectasia	D. Schindler et al	Full text	A*	B	C	A*	A*	A*	A*	A*	6
Serum IgD concentrations in patients with ataxia telangiectasia and with selective IgA deficiency	O. Sanal et al	Full text	B	A*	B	A*	A*	A*	A*	A*	6
Susceptibility to infections in patients with ataxia-telangiectasia (AT): A prospective follow-up study	S. Zielen et al	Full text (conf abstract)	B	A*	C	B	A*	A*, C	A*	A*	5
The Objective Assessment of Abnormal Eye-Movements in Infants and Young-Children	M. H. Jacobs et al	Full text	A*	B	A*	A*	None	A*, C	A*	A*	6
Transitional B cells and CD21low in patients with ataxia-telangiectasia	D. C. P. Bichuetti-Silva et al	Full text (conf abstract)	B	B	C	B	A*	A*	A*	A*	4
Unusual prevalence of Epstein-Barr virus early antigen (EBV-EA) antibodies in ataxia telangiectasia	J. L. Joncas et al	Full text	A*	A*	C	A*	A*	A*	A*	A*	7

Title	Author	Type of article	Case definition adequate	Representative of the cases	Selection of controls	Definition of controls	Comparability	Ascertainment of exposure	Same method for cases and controls	Non-response rate	Quality
Variant ataxia-telangiectasia presenting as primary-appearing dystonia in Canadian Mennonites.[Erratum appears in Neurology. 2012 Mar 27;78(13):1029 Note: Nakamura, T [corrected to Nakamura, K]]	R. Saunders-Pullman et al	Full text	A*	A*	A*	A*	A,B **	A,B **	A*	A*	10
Vitamin E serum levels are normal in ataxia telangiectasia (Louis-Bar disease)	C. Battisti et al	Full text	A*	A*	C	A*	A,B **	A*	A*	A*	8
DNA methylation and gene expression signatures are associated with ataxia-telangiectasia phenotype	McGrath-Morrow SA et al	Full text	A*	A*	A*	A*	A,B **	A*, C	A*	A*	9
Multiparametric cerebellar imaging and clinical phenotype in childhood ataxia telangiectasia	Dineen RA et al	Full text	A*	A*	A*	A*	A,B **	A*	A*	**B**	8
Selenium levels and glutathione peroxidase activity in patients with ataxia-telangiectasia: association with oxidative stress and lipid status biomarkers	Andrade IGA et al	Full text	A*	A*	**B**	A*	A*	A*, C	A*	A*	7
Free-Living Motor Activity Monitoring in Ataxia-Telangiectasia	Khan, NC; Pandey, V; Gajos, KZ; Gupta, AS	Full text	A*	B	C	A*	None	A*	A*	A*	5
Altered Cerebrospinal Fluid (CSF) in Children with Ataxia Telangiectasia	Woelke, S et al	Full text	A*	B	**B**	A*	None	A*	A*	A*	5
Normal Numbers of Stem Cell Memory T Cells Despite Strongly Reduced Naive T Cells Support	Weitering, TJ et al	Full text	A*	A*	C	A*	A*	A*, C	A*	A*	7

Title	Author	Type of article	Case definition adequate	Representative of the cases	Selection of controls	Definition of controls	Comparability	Ascertainment of exposure	Same method for cases and controls	Non-response rate	Quality
Intact Memory T Cell Compartment in Ataxia Telangiectasia											
Analysis of chromosomal aberrations and γH2A.X foci to identify radiation-sensitive ataxia-telangiectasia patients	Bucher M et al	Full text	A*	A*	**B**	A*	A,B **	A*, C	A*	A*	8
Immune competence and respiratory symptoms in patients with ataxia telangiectasia: A prospective follow-up study	Wölke S et al	Abstract	N/A	N/A	N/A	N/A	N/A	N/A	N/A	N/A	N/A
Variable Abnormalities in T and B Cell Subsets in Ataxia Telangiectasia	Shad, TM et al	Full text	A*	B	C	A*	A,B **	A*	A*	A*	7
Agreement of cardiovascular risk in ataxia-telangiectasia mutated heterozygotes and their children with Ataxia-telangiectasia	Barreto, TLN et al	Full text	A*	A*	A*	A*	A,B **	A*, C	A*	A*	9
The Role of Respiratory Viruses in Children with Ataxia-Telangiectasia	Mendez-Echevarria, A et al	Full text	A*	A*	A*	A*	A,B **	A*	A*	**B**	8

U/K – unknown, N/A – not applicable

S8 – Quality assessment Cohort studies

Title	Author	Type of article	Representativeness of the exposed cohort	Selection of the non-exposed cohort	Ascertainment of exposure	Demonstration outcome of interest not present at start of study	Comparability	Assessment of outcome	Was follow-up long enough?	Adequacy of follow-up	Stars	Quality (all)	Quality (comparability removed)
Cough ability measurements and recurrent respiratory symptoms in individuals with Ataxia Telangiectasia	D. Vilozni et al	Abstract only	A	unknown	A	B	A	B	Unknown - abstract only	Unknown - abstract only	4	Poor	Poor
FVC deterioration, airway obstruction determination, and life span in Ataxia telangiectasia	D. Vilozni et al	Full text	A	Unknown	A	B	A	B	A	A	6	Fair	Fair
Neuroimaging Findings and Repeat Neuroimaging Value in Pediatric Chronic Ataxia	M. S. Salman, Chodirker, B. N.,Bunge, M.	Full text	A	Unknown	A	B	A	B	Unable to determine	A	5	Fair	Fair
Alpha fetoprotein is increasing with age in ataxia-telangiectasia	A. Stray-Pedersen et al	Full text	A	N/A	A	A	N/A	B	A	A	6	Poor	Good

Title	Author	Type of article	Representativeness of the exposed cohort	Selection of the non-exposed cohort	Ascertainment of exposure	Demonstration outcome of interest not present at start of study	Comparability	Assessment of outcome	Was follow-up long enough?	Adequacy of follow-up	Stars	Quality (all)	Quality (comparability removed)
Alpha Fetoprotein Levels in Ataxia Telangiectasia as Related to Age, Disease Characteristics and Outcomes	Agress, A et al	Abstract only	A	N/A	A	B	N/A	B	Unknown - abstract only	A	4	Poor	Fair
Ataxia telangiectasia in Turkey: multisystem involvement of 91 patients	H. Akturk et al	Full text	A	N/A	A	B	N/A	B	A	A	5	Poor	Fair
Ataxia-Telangiectasia Clinical and Laboratory Features: Single Center Results	A. S. Sasihuseyinoglu et al	Abstract only	A	N/A	A	B	N/A	B	A	A	5	Poor	Fair
Ataxia-Telangiectasia in Costa Rica: Clinical, laboratory and genetic characteristics	O. Porras et al	Abstract only	A	N/A	A	A	N/A	B	A	A	4	Poor	Fair

Title	Author	Type of article	Representativeness of the exposed cohort	Selection of the non-exposed cohort	Ascertainment of exposure	Demonstration outcome of interest not present at start of study	Comparability	Assessment of outcome	Was follow-up long enough?	Adequacy of follow-up	Stars	Quality (all)	Quality (comparability removed)
Ataxia-telangiectasia: Clinical, laboratorial and mutational analysis of patients from a reference center for primary immunodeficiency	C. M. A. Jacob et al	Abstract only	A	N/A	A	B	N/A	B	B	A	4	Poor	Fair
Ataxia-telangiectasia: Immunodeficiency and survival	N. J. H. van Os et al	Full text	A	N/A	A	B	N/A	B	A	A	5	Poor	Fair
Cytogenetic and molecular studies of siblings with ataxia telangiectasia followed for 7 years	M. Renedo et al	Full text	A	N/A	A	A	N/A	B	A	A	6	Poor	Good
Deep Brain Stimulation in Rare Inherited Dystonias	I. Beaulieu-Boire et al	Full text	A	N/A	A	A	N/A	B	A	A	6	Poor	Good
Diagnostic considerations in ataxia-telangiectasia	J. M. G. Jason et al	Full text	A	N/A	A	A	N/A	B	A	A	6	Poor	Good

Title	Author	Type of article	Representativeness of the exposed cohort	Selection of the non-exposed cohort	Ascertainment of exposure	Demonstration outcome of interest not present at start of study	Comparability	Assessment of outcome	Was follow-up long enough?	Adequacy of follow-up	Stars	Quality (all)	Quality (comparability removed)
Do elevated serum IgM levels have to be included in probable diagnosis criteria of patients with ataxia-telangiectasia?	E. Azarsiz et al	Full text	A	N/A	A	B	N/A	B	A	A	5	Poor	Fair
Effects of inspiratory muscle training on lung volumes, respiratory muscle strength, and quality of life in patients with ataxia telangiectasia	E. Felix et al.	Abstract only	A	N/A	A	A	N/A	B	Unknown - abstract only	Unknown - abstract only	4	Poor	Poor
Endocrine abnormalities in ataxia telangiectasia: findings from a national cohort	A. Nissenkorn et al	Full text	A	N/A	A	B	N/A	B	B	A	4	Poor	Fair
Evaluation of infectious and non-infectious	S. Bazregari et al	Full text	A	N/A	A	B	N/A	B	Unable to determine	A	4	Poor	Fair

Title	Author	Type of article	Representativeness of the exposed cohort	Selection of the non-exposed cohort	Ascertainment of exposure	Demonstration outcome of interest not present at start of study	Comparability	Assessment of outcome	Was follow-up long enough?	Adequacy of follow-up	Stars	Quality (all)	Quality (comparability removed)
complications in patients with primary immunodeficiency													
Functional parameter measurements in children with ataxia telangiectasia	E. Shenhod et al	Full text	A	N/A	A	B	N/A	B	A	A	5	Poor	Fair
Longitudinal analysis of the neurological features of ataxia-telangiectasia	T. J. Jackson et al	Full text	A	N/A	A	B	N/A	B	A	A	5	Poor	Fair
Long-term nutritional and gastrointestinal aspects in patients with ataxia telangiectasia	A. Krauthammer et al	Full text	A	N/A	A	B	N/A	B	A	A	5	Poor	Fair
Microbiological surveillance in lung disease in ataxia telangiectasia	J. M. Bhatt et al	Full text	A	N/A	A	B	N/A	B	A	A	5	Poor	Fair

Title	Author	Type of article	Representativeness of the exposed cohort	Selection of the non-exposed cohort	Ascertainment of exposure	Demonstration outcome of interest not present at start of study	Comparability	Assessment of outcome	Was follow-up long enough?	Adequacy of follow-up	Stars	Quality (all)	Quality (comparability removed)
Morbidity and mortality from ataxia-telangiectasia are associated with ATM genotype	R. Micol et al	Full text	A	N/A	A	B	N/A	B	A	A	5	Poor	Fair
Neuromuscular abnormalities in ataxia telangiectasia: a clinical, electrophysiological and muscle ultrasound study	M. M. Verhagen et al	Full text	A	N/A	A	A	N/A	B	A	A	6	Poor	Good
Ocular findings in Norwegian patients with ataxia-telangiectasia: a 5 year prospective cohort study	R. Riise et al	Full text	A	N/A	A	A	N/A	B	A	A	6	Poor	Good
Phenotypic variations between affected siblings with ataxia-telangiectasia:	T. Morio et al	Full text	A	N/A	A	B	N/A	B	A	A	5	Poor	Fair

Title	Author	Type of article	Representativeness of the exposed cohort	Selection of the non-exposed cohort	Ascertainment of exposure	Demonstration outcome of interest not present at start of study	Comparability	Assessment of outcome	Was follow-up long enough?	Adequacy of follow-up	Stars	Quality (all)	Quality (comparability removed)
ataxia-telangiectasia in Japan													
Pre-emptive Allogeneic Hematopoietic Stem Cell Transplantation in Ataxia Telangiectasia	Bakhtiar, S et al	Full text	A	N/A	A	A	N/A	B	A	A	6	Poor	Good
Progressive Liver Disease in Patients With Ataxia Telangiectasia	Donath, H et al	Full text	A	N/A	A	B	N/A	B	A	A	5	Poor	Fair
Pulmonary function in adolescents with ataxia telangiectasia	S. McGrath-Morrow et al	Full text	A	N/A	A	A	N/A	B	A	A	6	Poor	Good
Safety and caregiver satisfaction with gastrostomy in patients with Ataxia Telangiectasia	M. A. Lefton-Greif et al	Full text	A	N/A	A	B	N/A	B	A	A	5	Poor	Fair

Title	Author	Type of article	Representativeness of the exposed cohort	Selection of the non-exposed cohort	Ascertainment of exposure	Demonstration outcome of interest not present at start of study	Comparability	Assessment of outcome	Was follow-up long enough?	Adequacy of follow-up	Stars	Quality (all)	Quality (comparability removed)
Survival probability in ataxia telangiectasia	T. O. Crawford et al	Full text	A	N/A	A	B	N/A	B	A	A	5	Poor	Fair
The feasibility and validity of forced spirometry in ataxia telangiectasia	D. Vilozni et al	Full text	A	N/A	A	A	N/A	A	A	A	6	Poor	Good
Variability of immunodeficiency associated with ataxia telangiectasia and clinical evolution in 12 affected patients	G. G. M. Claret Teruel, et al	Full text	A	N/A	A	B	N/A	B	A	A	5	Poor	Fair
Immune deficiency in Ataxia-Telangiectasia: a longitudinal study of 44 patients	C. Chopra et al	Full text	A	C	A	B	None	B	A	A	5	Poor	Fair
Immunodeficiency in ataxia telangiectasia is correlated strongly with the presence	E. R. Staples et al	Full text	A	A	A	B	None	B	A	A	6	Poor	Good

Title	Author	Type of article	Representativeness of the exposed cohort	Selection of the non-exposed cohort	Ascertainment of exposure	Demonstration outcome of interest not present at start of study	Comparability	Assessment of outcome	Was follow-up long enough?	Adequacy of follow-up	Stars	Quality (all)	Quality (comparability removed)
of two null mutations in the ataxia telangiectasia mutated gene													
Lymphoid tumours and breast cancer in ataxia telangiectasia; substantial protective effect of residual ATM kinase activity against childhood tumours	A. Reiman et al	Full text	A	A	A	B	None	B	A	A	6	Poor	Good
Clinical features and genetic analysis of Taiwanese patients with the hyper IgM syndrome phenotype	W. I. H. Lee et al	Abstract only	C	Unknown - abstract only	D	Unknown - abstract only	Unknown - abstract only	Unknown - abstract only	Unknown - abstract only	Unknown - abstract only	0	Poor	Poor
Clinical spectrum of ataxia-	M. M. Verhagen et al	Abstract only	A	A	A	B	Unknown - abstract only	B	Unknown - abstract only	A	5	Poor	Good

Title	Author	Type of article	Representativeness of the exposed cohort	Selection of the non-exposed cohort	Ascertainment of exposure	Demonstration outcome of interest not present at start of study	Comparability	Assessment of outcome	Was follow-up long enough?	Adequacy of follow-up	Stars	Quality (all)	Quality (comparability removed)
telangiectasia in adulthood													
Cranial MRI in ataxia-telangiectasia	F. Sardanelli et al	Abstract only	A	N/A	A	A	Unknown - abstract only	B	Unknown - abstract only	Unknown - abstract only	4	Poor	Poor
Effects of Inspiratory Muscle Training on Pulmonary Function in Patients With Ataxia-Telangiectasia	E. Felix et al	Abstract only	A	N/A	A	A	Unknown - abstract only	B	Unknown - abstract only	Unknown - abstract only	4	Poor	Poor
Hepatic disease in ataxia-telangiectasia, diagnosed in institutonacional de pediatria in Mexico City	R. Laurel et al	Abstract only	A	N/A	A	B	Unknown - abstract only	B	A	A	5	Poor	Fair
Immune status in ataxia telangiectasia	U. Datta et al	Abstract only	D	N/A	A	A	Unknown - abstract only	B	A	A	5	Poor	Fair
Immunological evaluation in Brazilian ataxia	A. S. Grumach et al	Abstract only	A	N/A	A	A	Unknown - abstract only	B	Unknown - abstract only	Unknown - abstract only	4	Poor	Poor

Title	Author	Type of article	Representativeness of the exposed cohort	Selection of the non-exposed cohort	Ascertainment of exposure	Demonstration outcome of interest not present at start of study	Comparability	Assessment of outcome	Was follow-up long enough?	Adequacy of follow-up	Stars	Quality (all)	Quality (comparability removed)
telangiectasia patients													
Late-breaking abstract: The Effect of lung volume deterioration on tidal volume, airway flows and life span in ataxia telangiectasia	D. Vilozni et al	Abstract only	A	N/A	A	B	Unknown - abstract only	B	A	A	5	Poor	Fair
Liver Disease in Pediatric Patients With Ataxia Telangiectasia: A Novel Report	B. Weiss et al	Abstract only	A	N/A	A	B	Unknown - abstract only	B	A	A	5	Poor	Fair
Longitudinal follow-up of lung function in ataxia telangiectasia	D. Vilozni et al	Abstract only	D	N/A	A	B	Unknown - abstract only	B	A	A	4	Poor	Poor
Neurocutaneous syndrome: A prospective study	R. S. Purkait et al	Full text	B	N/A	A	A	Unknown - abstract only	B	A	A	6	Poor	Good
Nutritional evaluation and food habbits in	B. T. Costa-Carvatho, et al	Abstract only	A	N/A	A	A	Unknown - abstract only	B	A	A	6	Poor	Good

Title	Author	Type of article	Representativeness of the exposed cohort	Selection of the non-exposed cohort	Ascertainment of exposure	Demonstration outcome of interest not present at start of study	Comparability	Assessment of outcome	Was follow-up long enough?	Adequacy of follow-up	Stars	Quality (all)	Quality (comparability removed)
patients with ataxia-telangiectasia (AT)													
Progressive liver disease in patients with ataxia telangiectasia	S. Woelke, et al.	Abstract only	A	N/A	A	B	Unknown - abstract only	B	A	A	5	Poor	Fair
Safety of live viral vaccines and retrospective review of immunologic status of 329 patients with ataxia telangiectasia	B. Uygungil et al	Abstract only	A	N/A	A	B	Unknown - abstract only	B	A	A	5	Poor	Fair
The clinical importance of performing forced spirometry in ataxia teleangiectasia	D. Vilozni et al	Abstract only	D	N/A	A	A	Unknown - abstract only	B	Unknown - abstract only	Unknown - abstract only	3	Poor	Poor
The microbiology of lung disease in ataxia telangiectasia (AT)	J. Bhatt et al	Abstract only	A	N/A	A	A	Unknown - abstract only	A	B	A	5	Poor	Good

Title	Author	Type of article	Representativeness of the exposed cohort	Selection of the non-exposed cohort	Ascertainment of exposure	Demonstration outcome of interest not present at start of study	Comparability	Assessment of outcome	Was follow-up long enough?	Adequacy of follow-up	Stars	Quality (all)	Quality (comparability removed)
Twenty-year follow-up of 160 patients with ataxia-telangiectasia	F. Ersoy et al	Abstract only	A	N/A	A	B	Unknown - abstract only	A	A	A	5	Poor	Fair
Ataxia Telangiectasia Diagnosed on Newborn Screening-Case Cohort of 5 Years' Experience	Mandola AB et al	Full text	A*	N/A	A*	A*	None	B*	A*	A*	5	Poor	Good
Functional parameter measurements in children with ataxia telangiectasia	Shenhod E et al	Full text	A*	N/A	A*	A*	None	B*	A*	A*	5	Poor	Good
A clinical profile of 100 patients with ataxia telangiectasia seen at a tertiary care center	Mahadevappa M et al	Full text	A*	N/A	A*	B	None	B*	A*	B*	3	Poor	Fair

Title	Author	Type of article	Representativeness of the exposed cohort	Selection of the non-exposed cohort	Ascertainment of exposure	Demonstration outcome of interest not present at start of study	Comparability	Assessment of outcome	Was follow-up long enough?	Adequacy of follow-up	Stars	Quality (all)	Quality (comparability removed)
Does the Hyper IgM Phenotype Affect Prognosis in Ataxia Telangiectasia?	Haskologlu, ZS et al	Full text	A*	N/A	A*	A*	None	B*	A*	A*	5	Poor	Good
Clinical and immunological presentation of ataxia-telangiectasia.	Oksana BOYARCHUK, et al	Full text	A*	N/A	A*	B	None	B*	A*	A*	4	Poor	Fair
Growth in ataxia telangiectasia	Natale VAI et al	Full text	A*	N/A	A*	B	None	B*	A*	A*	4	Poor	Fair
