# Supplementary material for: The natural history of ataxia-telangiectasia (A-T): A systematic review
Source: PLoS One. 2022 Mar 15;17(3):e0264177. doi: 10.1371/journal.pone.0264177 (PMC9049793; doi:10.1371/journal.pone.0264177)
Supplement: S2 File — (PDF) [file pone.0264177.s004.pdf]

## Figure and supplementary figure references

**3a – Family history of illness (non-A-T) -** Skin malignancy – 4 cases (4studies[74-77]), Other malignancy – 143 cases (30 studies[30, 33, 47, 57, 59, 72, 75-98]), Lymphoma – 7 cases (5 studies[75, 99-102]), Leukaemia – 9 cases (8 studies[22, 75, 76, 91, 92, 103-105]), Cognitive impairment – 4 cases (2 studies[106, 107]), Neurological conditions – 4 cases (3 studies[81, 108, 109]), Developmental difficulties – 3 cases (2 studies[107, 110]), Death at an early age - 3 cases (1 study[33]), Other conditions – 24 cases (8 studies[109-117]). Immune difficulties – 2 cases (2 studies[85, 114]).

**3b – Presenting signs and symptoms of A-T**

Eye signs – 7 cases (6 studies[71, 212, 214, 268, 317, 468]), Developmental difficulties – 30 cases (28 studies[99, 110, 124, 126, 129, 150, 154, 166, 199, 204, 212, 219, 239, 246, 272, 391, 405, 423, 455, 461, 473, 479, 483, 516-521]), Abnormal gait – 1160 cases (188 studies[17, 18, 22, 24, 33, 38, 42, 46, 49, 57, 61, 63, 72, 74, 76, 84, 87, 89, 99, 101, 103, 106-110, 113, 115, 116, 119, 120, 123, 125, 129, 131, 132, 137-143, 147, 148, 152, 154, 159, 161, 164, 172, 178, 180, 181, 183, 184, 190, 193, 195, 198, 199, 204, 208, 211, 213, 214, 216, 219, 221, 222, 230, 231, 234, 236, 238, 240, 242, 244, 245, 248, 255, 257, 259-266, 274, 275, 277, 281, 284, 285, 287-289, 294, 299, 305, 310, 315, 317, 319, 323, 325, 328, 331, 345, 347, 354, 355, 372, 379, 382, 387, 388, 392, 394, 395, 397-400, 403-406, 409, 411, 412, 417, 426, 431, 433, 434, 447, 448, 455, 457, 458, 464, 466, 469, 472-474, 478, 482, 483, 485, 486, 488, 489, 491, 493, 499-501, 504, 513, 518, 519, 522-544]), Tremor – 16 cases (11 studies[27, 31, 35, 38, 52, 72, 103, 263, 338, 380, 528]), Movement disorders – 70 cases (30 studies[27, 28, 35, 41, 46-49, 54, 59, 61, 62, 66, 86, 123, 143, 172, 214, 242, 251, 273, 288, 396, 417, 424, 526, 537, 539, 545, 546]), Other co-ordination difficulties – 105 cases (48 studies[30, 61, 66, 86, 89, 109, 111, 120, 139, 161, 190, 199, 206, 223, 230, 233, 242, 252, 261, 262, 266, 273, 288, 298, 324, 335, 347, 372, 383, 384, 387, 390, 391, 398, 415, 417, 428, 448, 451, 458, 461, 484, 526, 528, 534, 546-548]), Other neurological sign/symptom – 131 cases (33 studies[18, 27, 33, 52, 80, 101, 107-109, 111, 124, 159, 166, 198, 213, 219, 222, 237, 261, 287, 288, 317, 340, 385, 415, 417, 447, 486, 534, 544, 549, 550]), Speech difficulties – 116 cases (23 studies[33, 65, 99, 103, 107, 138, 139, 143, 216, 240, 265, 347, 372, 395, 431, 448, 476, 479, 505, 528, 534, 539, 544]), Other/unspecified malignancy – 6 cases (4 studies[66, 229, 423, 522]), Leukaemia – 19 cases (12 studies[39, 66, 82, 102, 154, 337, 404, 464, 465, 496, 537, 551]), Lymphoma – 10 cases (10 studies[148, 169, 275, 337, 359, 387, 429, 492, 552,

[553]), Telangiectasia – 59 cases (33 studies[17, 76, 87, 115, 124, 142, 154, 178, 198, 221, 224, 228, 237, 259, 275, 281, 285, 289, 317, 347, 355, 382, 385, 426, 431, 455, 485, 501, 513, 522, 535, 540, 554]), Recurrent infections – 227 cases (68 studies[21, 22, 24, 53, 83, 87, 99, 115, 116, 121, 122, 128, 129, 131, 133, 135, 137, 139, 142, 143, 147, 193, 194, 196, 198, 199, 211, 228, 231, 238, 239, 241, 247, 249, 255, 269, 270, 276, 294, 331, 345, 355, 373, 414, 416, 418, 425, 435, 439, 450, 451, 458, 467, 470, 483, 487, 497, 501, 502, 505, 513, 522, 526, 531, 534, 548, 549, 555, 556], Other infections – 6 cases (6 studies[203, 249, 294, 419, 430, 541]), Haematological abnormalities – 16 cases (10 studies[17, 24, 134, 197, 203, 289, 294, 359, 419, 435]), Gastrointestinal sign/symptom – 16 cases (16 studies[17, 24, 53, 99, 124, 134, 187, 194, 197, 203, 208, 259, 289, 294, 416, 419]), Respiratory sign/symptom – 3 cases (3 studies[92, 187, 491]), Immunological abnormaility – 3 cases (3 studies[203, 243, 491]), Poor growth/failure to thrive – 13 cases (13 studies[197, 208, 228, 246, 260, 263, 287, 425, 439, 450, 458, 483, 533]), Other sign/symptom – 43 cases (33 studies[18, 24, 33, 52, 65, 66, 94, 95, 101, 104, 106, 129, 203, 208, 211, 241, 252, 259, 264, 416, 443, 460, 476, 479, 480, 506, 517, 544, 557-561]), Dermatological sign/symptom – 14 cases (12 studies[165, 197, 246, 247, 285, 351, 418, 452, 470, 481, 533, 534]).

**4a – Ataxia and mobility - Cerebellar gait ataxia** - 3223 cases (405 studies[3, 16-19, 21, 22, 24, 27, 29, 31, 33, 36-38, 74, 75, 78, 80, 86-89, 99, 101, 106-109, 111-113, 115, 116, 118-126, 128, 129, 131, 133-135, 139-141, 143-145, 147, 148, 151, 155, 158, 159, 165, 166, 222-225, 228-231, 233, 234, 237, 239-248, 254-256, 281, 284, 285, 287-290, 294, 298-300, 302-305, 307, 310, 316, 317, 319, 323-326, 369, 372, 374, 379, 380, 382-384, 387-391, 400, 401, 407, 409-411, 414-416, 423, 425, 426, 428, 429, 431-435, 438-440, 443-445, 447, 448, 450, 451, 455-458, 464, 510, 518, 523-528, 530-533, 545-547, 549, 555, 562-602] [24, 42, 43, 46-50, 52, 53, 57, 58, 63, 66-68, 71, 72, 76, 77, 81, 84, 93-96, 100, 103, 105, 108, 110, 172, 174, 175, 177-181, 183, 184, 186, 187, 189-194, 196, 199, 200, 202-208, 210-213, 216-221, 250, 257, 258, 260, 262-276, 278, 331, 332, 335, 338-340, 342, 345-350, 353-355, 358, 360, 361, 363, 365, 366, 368, 378, 392-396, 398, 403-406, 467-470, 472, 473, 476-478, 481-486, 488-492, 494, 500-505, 513, 519-521, 534-538, 540, 541, 556, 603-631]), **Truncal ataxia** - 357 cases (69 studies[17, 33, 48, 61, 66, 75, 87, 95, 99, 100, 111, 123, 138-140, 190, 202, 206, 208, 211, 214, 221-223, 225, 230, 231, 233, 234, 236, 238, 242, 244, 254, 257, 262-264, 266, 282, 287, 324, 331, 338, 340, 342, 372, 384, 385, 392, 404, 405, 415-417, 428, 443, 451, 484, 488, 524, 534, 544, 573, 582, 583, 606, 620, 632, 633]). **Limb ataxia** - 163 cases (31 studies[27, 33, 42, 46, 63, 123, 158, 159, 190, 213, 223-225, 257, 261, 266, 271, 273, 275, 290, 325, 340, 348, 368, 372, 380, 383, 392, 398, 416, 519]), **Wheelchair bound** – 769 cases (112 studies[3, 19, 24, 25, 27, 31, 33, 35, 36, 38, 46, 49, 52, 57, 58, 66,

76, 79, 87, 99, 109, 115, 118, 120, 123, 125-127, 131, 132, 139, 140, 154, 161, 166, 168, 175, 190, 195, 204, 208, 213, 225, 226, 230, 240, 242, 248, 261, 265, 268, 275, 283, 287, 288, 310, 317, 325, 335, 342, 345-347, 354, 362, 368, 372, 373, 375, 391, 392, 394, 395, 438, 448, 449, 483, 484, 490, 494, 498, 503, 513, 523-525, 528, 530, 536, 537, 547, 573, 580, 585, 597, 600, 609, 614, 617, 619, 620, 625, 634-643]),

**Requiring support to walk** - 119 cases (44 studies[3, 16, 33, 36, 42, 46, 47, 57, 63, 68, 84, 109, 120, 122, 126, 140, 165, 212, 215, 225, 231, 234, 237, 238, 248, 272, 276, 299, 331, 372, 401, 406, 407, 426, 445, 472, 476, 606, 619, 620, 635, 637, 643, 644]), **Bed-bound** - 17 cases (6 studies[33, 87, 224, 331, 449, 492]).

**4c – Eye manifestations**

Oculomotor apraxia - 1213 cases (89 studies[22, 35-37, 49, 52, 68, 75, 76, 84, 87, 99, 108, 109, 122, 126, 131, 138, 139, 141, 143, 169, 174-176, 180, 181, 183, 191, 199, 202, 211-213, 219, 225, 230, 231, 238, 245, 251, 257, 260, 261, 265, 268, 269, 271, 273, 275, 278, 287, 299, 303, 317, 326, 350, 368, 369, 372, 388, 394, 405, 409, 411, 429, 449, 482, 503, 519-521, 523, 525, 527-529, 537, 539, 563, 564, 571, 574, 585, 601, 604, 610, 623]), Strabismus – 48 cases (18 studies[17, 23, 41, 55, 59, 123, 194, 199, 214, 265, 345, 372, 445, 501, 518, 614, 645, 646]), Abnormal pursuit – 240 cases (21 studies[17, 47, 61, 119, 123, 143, 159, 213, 216, 310, 344, 398, 476, 490, 503, 537, 596, 611, 645-647]), Nystagmus – 346 cases (104 studies[17, 18, 23, 37, 42, 52, 53, 63, 66, 72, 76, 80, 87, 95, 103, 108, 112, 119, 120, 122-124, 126, 138, 143, 147, 159, 165, 174, 177, 180, 181, 190, 191, 194, 202, 206, 212-214, 217, 221, 224, 225, 230, 231, 233, 237, 257, 262, 265, 268, 271, 272, 275, 287, 288, 290, 298, 299, 323, 325, 326, 328, 331, 344, 345, 360, 363, 366, 368, 372, 380, 388-390, 394, 400, 401, 409, 435, 445, 448, 472, 495, 501, 518, 519, 524, 527, 530, 534, 538, 574, 593, 596, 609, 645-651]), Abnormal saccades – 282 cases (38 studies[17, 33, 47, 71, 119, 123, 143, 159, 180, 202, 213, 216, 223, 228, 233, 234, 237, 261, 262, 273, 290, 310, 344, 392, 396, 398, 402, 434, 443, 476, 503, 537, 583, 609, 645, 646, 649, 652]).

**4e – Other neurological manifestations**

Seizures – 31 cases (13 studies[75, 76, 87, 108, 109, 123, 124, 198, 211, 288, 368, 407, 641]), Abnormal sensory examination – 14 cases (39 studies[22, 27, 33, 46, 49, 52, 53, 55, 61, 63, 72, 118, 121, 123, 159, 181, 198, 213, 230, 251, 287, 299, 328, 345, 358, 368, 372, 392, 409, 410, 448, 449, 490, 503, 513, 519, 528, 585, 653]), Abnormal light touch - 12 cases (3 studies[72, 409, 585]), Abnormal pinprick sensation - 4 cases (4 studies[33, 72, 159, 409]), Abnormal vibration - 16 cases (14 studies [33, 55, 63, 72, 121, 123, 159, 328, 372, 392, 409, 448, 503, 528]),

Abnormal proprioception – 58 cases (13 studies[33, 49, 55, 72, 123, 230, 251, 287, 345, 372, 392, 490, 585]), Peripheral neuropathy – 103 cases (14 studies[22, 46, 52, 61, 123, 181, 198, 287, 358, 410, 449, 513, 518, 653]), Drooling – 179 cases (36 studies[59, 63, 66, 84, 95, 108, 120, 123, 138, 159, 175, 181, 222, 231, 234, 238, 265, 287, 298, 310, 324, 325, 331, 345, 366, 367, 379, 400, 415, 417, 469, 494, 544, 549, 590, 654]), Muscle atrophy – 57 cases (27 studies[16, 33, 38, 49, 53, 72, 77, 112, 118, 120, 123, 139, 180, 190, 225, 237, 281, 323, 338, 345, 380, 391, 407, 503, 527, 655, 656]), Contractures – 16 cases (5 studies[231, 248, 287, 403, 449])

**5a – Tone, weakness and reflexes**

Hyporeflexia in lower limbs – 74 cases (53 studies[17, 33, 53, 55, 72, 81, 91, 99, 113, 122, 126, 141, 165, 181, 190, 217, 222, 224, 228-230, 236, 238, 240, 242, 248, 251, 266, 290, 310, 331, 368, 372, 384, 385, 388, 401, 405, 409, 411, 445, 448, 472, 495, 503, 519, 528, 530, 536, 574, 604, 606, 651]), Brisk reflexes in lower limbs – 10 cases (10 studies[18, 47, 272, 372, 403, 453, 469, 476, 544, 603]), Hyporeflexia in upper limbs – 48 cases (43 studies[17, 33, 53, 72, 81, 99, 122, 126, 165, 181, 190, 217, 222, 224, 228-230, 236-238, 240, 242, 248, 266, 290, 310, 384, 385, 388, 401, 405, 411, 445, 448, 472, 495, 503, 519, 530, 536, 574, 606, 651]), Brisk reflexes in upper limb – 8 cases (8 studies[18, 47, 272, 403, 453, 469, 544, 603]).

**Muscle tone and weakness** – Muscle hypotonia - 217cases (62 studies[41, 49, 66, 95, 99, 101, 111, 116, 120, 122, 126, 135, 148, 166, 169, 174, 193, 194, 213, 217, 221-223, 225, 229-231, 234, 237, 238, 240, 248, 260-262, 268, 269, 285, 287, 290, 298, 299, 310, 325, 331, 340, 387, 403, 405, 407, 434, 447, 472, 473, 516, 518, 520, 521, 524, 534, 549, 574]), Muscle hypertonia – 10 cases (9 studies[176, 254, 265, 272, 287, 299, 323, 335, 407, 469, 476]), Distal muscle weakness – 16 cases (10 studies[33, 38, 41, 123, 180, 231, 323, 338, 519, 563]), Proximal muscle weakness – 6 cases (4 studies[110, 139, 384, 544]), Generalised muscle weakness – 29 cases (20 studies[53, 107, 108, 111, 118, 181, 194, 230, 266, 285, 347, 372, 380, 449, 472, 499, 518, 657-659]), Unspecified hyporeflexia - 286 cases (45 studies[33, 38, 41, 49, 63, 106, 108, 109, 118-120, 123, 129, 138, 139, 143, 159, 191, 212, 213, 220, 221, 225, 231, 232, 234, 257, 261, 265, 287, 325, 328, 335, 338, 345, 366, 400, 407, 524, 527, 585, 604, 607, 609, 656]), Unspecified hyperreflexia - 12 cases (4 studies[66, 231, 262, 407]).

**5b – Movement disorder manifestations**

Choreoathetosis – 304 cases (59 studies[27, 33, 35-37, 42, 48, 52, 58, 59, 63, 75, 77, 87, 108, 118, 119, 140, 141, 143, 145, 152, 181, 190, 191, 194, 219, 225, 232, 236, 242, 244, 245, 251, 257, 285, 299, 325, 332, 335, 350, 368, 372, 384, 391, 407, 417, 424, 490, 520, 521, 524, 565, 598, 610, 623, 633, 660, 661]), Chorea only – 152 cases (23 studies[35, 41, 61, 66, 80, 81, 111, 123, 138, 159, 168, 216, 227, 231, 237, 287, 288, 346, 398, 469, 537, 568, 607]), Athetosis only – 111 cases (18 studies[33, 35, 59, 86, 223, 224, 242, 248, 252, 287, 345, 372, 400, 401, 403, 406, 434, 607]), Myoclonus – 95 cases (38 studies[27, 33, 47, 48, 52, 66, 74, 76, 86, 91, 94, 105, 108, 123, 141, 181, 190, 238, 242, 251, 254, 257, 261, 265, 287, 325, 335, 345, 366, 372, 392, 472, 476, 519, 537, 539, 590, 656]), Dystonia – 282 cases (80 studies[27, 28, 31, 35, 36, 41, 47, 49, 50, 52, 54, 57-59, 61-63, 65, 66, 72, 74, 81, 89, 94, 103, 123, 135, 151, 172, 190, 199, 208, 213, 216, 219, 227, 234, 237, 244, 245, 247, 251, 262, 273, 287, 350, 363, 367, 368, 375, 381, 385, 395, 405, 411, 434, 445, 449, 453, 476, 516, 519-521, 537, 539, 545, 546, 571, 585, 590, 606, 607, 621, 622, 660-664]), Bradykinesia – 88 cases (7 studies[31, 363, 529, 537, 565, 606, 607]), Hyperkinesia – 43 cases (3 studies[406, 537, 606]), Tremor – 363 cases (80 studies[27, 30, 31, 35, 36, 38, 42, 48, 52, 53, 61, 66, 69, 72, 76, 99, 103, 108, 111, 122, 124, 126, 135, 139, 140, 159, 174, 181, 190, 199, 211, 212, 217, 222, 227, 230, 231, 234, 238, 242, 245, 260, 263, 275, 276, 287, 338, 342, 345, 363, 372, 380, 381, 397, 401, 405, 406, 416, 434, 448, 472, 492, 518, 524, 528, 538, 539, 547, 585, 586, 590, 597, 606, 607, 619, 620, 625, 629, 665, 666]), Fasiculations – 6 cases (5 studies[33, 118, 139, 248, 656]).

**5d – Cerebellar signs**

Apraxia – 7 cases (1 study[342]), Romberg's positive – 11 cases (7 studies[53, 184, 213, 319, 384, 387, 501]), Undefined cerebellar signs – 14 cases (13 studies[27, 69, 108, 110, 165, 193, 251, 255, 263, 272, 385, 423, 519]), Dyssynergy – 17 cases (6 studies[129, 174, 180, 335, 400, 572]), Dysmetria – 236 cases (48 studies[33, 47, 55, 66, 84, 95, 106, 108, 116, 120, 122, 126, 135, 138, 175, 191, 217, 221, 223, 230, 231, 234, 236, 260, 310, 335, 347, 388, 400, 401, 405, 407, 409, 445, 448, 476, 490, 501, 524, 528, 529, 537, 574, 586, 591, 596, 620, 621]), Dysdiadokinesia – 59 cases (31 studies[47, 84, 103, 108, 116, 120, 122, 124, 126, 129, 138, 159, 174, 184, 217, 221, 223, 231, 234, 236, 265, 319, 331, 345, 397, 401, 407, 443, 448, 476, 528]), Fine motor co-ordination difficulties – 9 cases (7 studies[17, 38, 63, 275, 282, 379, 667]), Undefined co-ordination difficulties – 17 cases (17 studies[111, 148, 181, 231, 237, 238, 273, 288, 319, 372, 373, 383, 428, 444, 448, 543, 625]), Abnormal finger-nose test – 16 cases (8 studies[110, 196, 212, 242, 310, 387, 425, 547, 651]).

**8c – Neuroimaging findings -** Abnormal neuroimaging – 547 cases (155 studies[17, 18, 25, 27, 33, 35, 38, 42, 49, 54, 61, 63, 67, 72, 76, 87, 94, 101, 103, 108-110, 119-123, 125, 132, 138, 139, 143-145, 147, 148, 151, 152, 159, 172, 175, 177, 180, 184, 190, 191, 193, 207, 208, 211-214, 216, 217, 220, 225, 226, 231, 236-239, 251, 256, 257, 259, 263, 265, 266, 276, 290, 298, 299, 307, 328, 338, 342, 345, 347, 354, 355, 358, 368, 373, 378, 387-389, 392, 395-398, 405, 409, 412, 417, 423, 426, 431, 434, 442-445, 458, 464, 467, 468, 476, 478, 485, 492, 499, 501-503, 528, 530, 534, 536, 540, 557, 563, 585, 586, 591, 593, 599, 609, 616, 620, 627, 631, 632, 641, 651, 661, 663, 668-682] ), Cerebellar atrophy/hypoplasia– 499 cases (144 studies[17, 18, 25, 27, 33, 35, 38, 42, 49, 54, 63, 67, 72, 76, 87, 94, 101, 103, 108-110, 119-123, 125, 132, 138, 139, 143-145, 147, 148, 152, 159, 172, 175, 177, 180, 184, 190, 191, 193, 207, 208, 211-214, 216, 217, 220, 225, 226, 231, 236-239, 251, 256, 257, 259, 263, 265, 266, 276, 290, 298, 299, 307, 328, 338, 342, 345, 347, 354, 355, 358, 368, 373, 378, 387, 388, 392, 395-398, 405, 409, 412, 417, 423, 426, 431, 434, 443-445, 458, 464, 467, 468, 476, 478, 485, 492, 499, 501-503, 528, 530, 534, 536, 540, 563, 585, 586, 591, 593, 599, 609, 616, 620, 627, 631, 641, 651, 661, 663, 669, 672-678, 680, 681]), Increased subarachnoid space – 3 cases (2 studies[263, 673]), Cerebral atrophy – 4 cases (3 studies[123, 211, 458]), Atrophy of corpus nucleus caudate – 1 case (1 study[35]), White matter changes – 12 cases (4 studies[35, 442, 632, 661]), Cerebral microbleeds – 3 cases (2 studies[35, 444]), Basal ganglia changes – 9 cases (2 studies[661, 679]), Myelin atrophy – 8 cases (1 study[661]), Pontine atrophy – 10 cases (2 studies[641, 661]), Cerebellar calcified spots – 1 case (1 study[121]), Multiple infarctions – 1 case (1 study[354]), White matter dysmyelination – 1 case (1 study[671]), Megalencephaly – 3 cases (1 study[677]), Chiari 1 malformation – 1 case (1 study[677]), Cerebral mass lesion – 4 cases (2 studies[677, 680]), Capillary telangiectasia – 4 cases (1 study[680]), Vascular abnormality – 4 cases (4 studies[76, 121, 389, 670]).

**5e – Immunoglobulins**

Low IgA – 1556 cases (282 studies[19, 21, 23, 24, 28, 35, 39, 40, 43, 75, 78, 80, 87, 90, 99, 101, 107, 113, 114, 118-121, 123, 124, 128-134, 138, 139, 147, 148, 150, 152, 154, 158, 160, 165, 174, 214, 222, 224, 225, 233, 236, 239, 241, 243, 246-248, 250, 252, 256, 258-260, 278, 281, 283, 285, 287-289, 294, 296, 298, 299, 302, 303, 305, 310, 316-318, 321, 329-331, 333-336, 339, 340, 369, 374-376, 384, 389, 404, 407, 409, 410, 415, 416, 418, 420, 425, 426, 428, 431-436, 438, 439, 445, 448-451, 458, 461, 464, 470, 510, 511, 513, 515, 523, 525, 527, 529, 532-535, 546, 549, 567, 570, 575, 579, 582, 591, 594, 595, 598-600, 603, 609, 657, 685-707][49, 51-53, 59, 66, 68, 76, 83, 84, 95, 100, 110, 178, 180, 181, 183, 184, 190, 191, 193, 194, 196, 199-201, 203-208, 210, 211, 213, 216, 218, 219, 221, 261, 262, 264, 266, 267, 269-271,

274, 275, 345-347, 353-355, 358, 362, 364, 378, 395, 396, 398, 406, 478, 480, 484, 485, 495, 501, 516, 538, 540, 541, 552, 556, 622, 627, 628, 633, 639, 640, 642, 708-722]), Normal IgA – 430 cases (104 studies[19, 24, 27, 35, 38-42, 51, 62, 63, 65, 68, 90, 95, 103, 118-120, 123, 124, 131, 132, 135, 138, 152, 159, 174, 191, 197, 199, 221, 225, 229, 238, 249, 251, 263, 266-268, 275, 276, 278, 280, 287, 294, 299, 305, 316, 330, 331, 333, 338, 346, 360-363, 369, 376, 381, 383, 388, 390-392, 407, 411, 419, 423, 440, 449, 467, 469, 476, 524, 528, 530, 536, 540, 545, 555, 581, 589, 598, 622, 642, 658, 686-688, 691, 692, 696, 697, 702, 704, 706, 708, 712, 714, 723, 724]), High IgA – 56 cases (22 studies[19, 24, 33, 40, 51, 75, 76, 91, 131, 174, 217, 221, 237, 272, 278, 346, 361, 385, 475, 502, 696, 725]), Low IgG –418 cases (127 studies[19, 21, 24, 35, 39, 40, 42, 43, 51, 52, 76, 78, 84, 87, 95, 105, 107, 114, 120, 128, 132-134, 150, 154, 158, 160, 165, 174, 181, 199, 201, 203, 205-208, 210, 211, 213, 219, 221, 225, 226, 228, 236, 237, 239, 243, 246, 247, 249, 252, 259, 264, 269, 271, 274, 275, 289, 294, 298, 305, 331, 333, 335, 339, 346, 361, 364, 369, 376, 391, 395, 407, 416, 418, 425, 428, 433, 439, 451, 456, 458, 461, 464, 470, 492, 497, 501, 502, 516, 523, 525, 528-530, 533, 534, 536, 540, 541, 546, 556, 567, 580, 582, 583, 595, 599, 627, 628, 642, 658, 686, 687, 693, 695, 699, 706, 709, 712, 713, 717, 718, 722, 726]), Normal IgG – 576 cases (102 studies[19, 23, 24, 27, 35, 38-41, 51, 53, 62, 63, 65, 80, 83, 90, 91, 95, 99, 103, 110, 119, 120, 123, 124, 132, 135, 138, 157, 159, 168, 174, 199, 205, 217, 218, 221, 225, 229, 238, 241, 248, 251, 258, 263, 266, 268, 272, 283, 288, 312, 333, 335, 346, 360, 361, 363, 369, 375, 381, 384, 390, 392, 396, 404, 407, 411, 419, 423, 426, 435, 440, 448, 449, 455, 467, 469, 476, 480, 484, 528, 532, 538, 555, 581, 594, 599, 625, 642, 686, 699, 705-707, 712, 720, 723, 724, 727, 728]), High IgG – 74 cases (22 studies[24, 33, 35, 40, 51, 76, 148, 174, 196, 197, 218, 222, 224, 266, 288, 331, 333, 346, 385, 524, 699, 705]), Low IgG1 – 11 cases (7 studies[39, 43, 174, 458, 685, 699, 719]), Normal IgG1 – 47 cases (11 studies[19, 138, 174, 222, 247, 338, 381, 390, 484, 696, 704]), High IgG1 – 15 cases (5 studies[19, 138, 174, 704, 725]), Low IgG2 – 290 cases (51 studies[19, 24, 39, 40, 43, 51, 52, 83, 87, 90, 130, 132, 138, 168, 169, 174, 180, 191, 197, 204, 247, 269, 270, 287, 303, 317, 390, 426, 429, 443, 449, 451, 458, 484, 513, 523, 531, 555, 570, 631, 639, 642, 685, 696, 699, 704-706, 711, 719, 727]), Normal IgG2 – 91 cases (19 studies[19, 24, 40, 51, 90, 132, 138, 174, 194, 222, 287, 338, 381, 449, 642, 685, 696, 704, 727]), High IgG2 – 7 cases (1 study[51]), Low IgG3 – 46 cases (21 studies[19, 39, 43, 51, 138, 174, 191, 194, 197, 247, 267, 269, 338, 381, 390, 443, 458, 484, 513, 570, 631), Normal IgG3 – 61 cases (12 studies{Nowak-Wegrzyn, 1998 #51640, 696, 699, 704, 705, 719]), High IgG3 – 2 cases (2 studies[51, 174]), Low IgG4 – 88 cases (22 studies[43, 83, 138, 174, 204, 214, 222, 270, 426, 429, 443, 451, 552, 555, 631, 639, 696, 699, 704, 706, 711, 719]), Normal IgG4 – 23 cases (10 studies[19, 138, 174, 194, 338, 381, 390, 484, 696, 704] ), High IgG4 – 0 cases, Low IgM – 25 cases (18 studies[19, 40, 42, 114, 213, 241, 247, 316, 364, 369, 407, 425, 480, 495, 524, 533, 595, 657]), Normal IgM – 560 cases (100 studies[19, 21, 23, 24, 27, 35, 38-41, 51, 53, 62, 65, 80, 84, 91, 95, 103, 110, 119, 120, 123, 124, 132, 135, 138, 159, 165, 174, 197, 205, 207, 211, 217, 218, 221, 229, 236, 238, 243, 246, 248, 252, 258, 263, 264, 267, 269, 270, 283, 287, 288, 294, 298, 305,

330, 331, 333, 335, 338, 346, 360-363, 375, 381, 384, 391, 396, 407, 411, 419, 423, 440, 455, 467, 469, 470, 476, 484, 528, 530, 536, 538, 540, 555, 594, 598, 599, 642, 686, 695, 696, 699, 704, 706, 709, 724]), High IgM – 471 cases (108 studies[19, 24, 33, 35, 39, 40, 51, 63, 74, 76, 78, 83, 87, 95, 105, 107, 120, 128, 130, 132-134, 148, 151, 154, 160, 174, 196, 199, 201, 203, 205, 206, 208, 210, 211, 218, 219, 221, 222, 224, 228, 237, 239, 249, 259, 266, 267, 272, 289, 294, 331, 333, 335, 338, 346, 361, 385, 390, 404, 407, 414, 416, 418, 426, 428, 433, 435, 448-450, 456, 457, 470, 500-502, 510, 524, 532, 534, 550, 552, 567, 577, 582, 602, 640, 642, 686, 687, 694, 696, 699, 704, 707, 710, 717, 718, 720, 722, 725, 728-733]), Normal IgD – 10 cases (10 studies[203, 375, 381, 411, 419, 423, 495, 555, 728, 734]), High IgD – 4 cases (2 studies[710, 734]), Low IgE – 243 cases (53 studies[35, 51, 66, 76, 83, 113, 121, 129, 138, 152, 178, 180, 204-206, 211, 213, 216, 221, 222, 264, 278, 318, 335, 336, 340, 376, 391, 398, 418, 420, 428, 440, 445, 448, 469, 476, 484, 493, 525, 534, 549, 567, 575, 580, 582, 591, 658, 689, 699, 702, 704, 710, 735]), Normal IgE – 163 cases (48 studies[27, 33, 35, 38, 51, 65, 119, 148, 165, 197, 203, 205, 207, 208, 211, 221, 229, 238, 252, 259, 266, 269, 305, 335, 338, 346, 358, 360, 362, 375, 376, 381, 383, 390, 407, 411, 419, 423, 433, 502, 530, 536, 555, 589, 599, 696, 702, 704]),), High IgE – 18 cases (9 studies[33, 76, 87, 100, 152, 158, 194, 338, 407]).

**5f – Immunoglobulin replacement and prophylactic antibiotics**

Immunoglobulin replacement – 819 cases (145 studies[19, 21, 24, 39, 40, 49, 51, 52, 76, 78, 83, 84, 87, 94, 95, 105, 107, 117, 120, 127, 128, 132-135, 142, 154, 175, 177, 185, 195-197, 203, 206, 208, 211, 214, 219, 221, 226, 228, 236, 241, 243, 247, 249, 259, 260, 269, 274, 278, 289, 294, 315, 317, 327, 331, 337, 340, 357, 359, 360, 364, 366, 404, 410, 416, 418, 427, 428, 436, 438, 439, 451, 455, 457, 462, 470, 487, 491, 500-502, 507, 513, 515, 517, 523, 530, 533, 534, 536, 550, 552, 556, 580, 588, 594, 599, 614, 615, 617, 628, 631, 632, 634, 639, 640, 642, 667, 699, 706, 711, 715, 718, 722, 726, 736-762]), No immunoglobulin replacement – 530 cases (22 studies[19, 24, 35, 39, 40, 51, 142, 175, 233, 315, 373, 391, 425, 449, 580, 594, 704, 715, 743, 747, 749, 754]), Prophylactic antibitoics – 332 cases (50 studies[24, 39, 40, 52, 76, 83, 84, 87, 120, 128, 134, 135, 154, 196, 203, 211, 219, 221, 228, 230, 241, 243, 247, 259, 264, 274, 278, 294, 360, 364, 451, 457, 462, 470, 500-502, 507, 515, 517, 523, 550, 555, 588, 617, 699, 704, 749, 761, 763]), No prophylactic antibiotics – 121 cases (7 studies[24, 40, 52, 236, 396, 449, 749])

**6b – Recurrent infections**

Recurrent infections – 1326 cases including pneumonia/LRTI - (269 studies[19, 21, 39, 40, 43, 49, 53, 59, 63, 83, 87, 99, 108-111, 113, 114, 116, 118, 120, 121, 124, 126-128, 132, 135, 141, 142, 147, 148, 155, 158, 165, 168, 174, 180, 181, 185, 190, 191, 198, 201, 204, 214, 222, 225, 228, 231, 236, 241, 247, 248, 252, 263, 264, 267, 269-271, 276, 278, 281, 284, 285, 287, 288, 294, 298, 299, 304, 305, 310, 317, 319, 331, 332, 339, 340, 345, 353, 355, 360, 369, 376, 379, 382, 384, 385, 387, 395, 400, 401, 403, 407, 410, 411, 414, 416, 418, 419, 428, 429, 435, 436, 439, 440, 443, 450, 455, 456, 462, 473, 483, 489, 497, 501, 502, 515, 524, 525, 527, 529, 531, 534, 535, 538, 541-543, 546, 547, 549, 555, 556, 558, 567, 571, 573, 582, 588, 594, 595, 598, 614, 618, 627, 639, 654, 657, 673, 689, 702, 710, 764-768][22-24, 28, 33, 37, 38, 42, 46, 66, 76, 78, 84, 104, 112, 119, 122, 129, 131, 133, 134, 139, 145, 152, 154, 160, 168, 169, 175, 194, 196, 199, 205, 206, 210, 213, 217-221, 226, 232, 234, 238-240, 242, 244, 249, 251, 255-257, 260, 265, 270, 272-275, 302, 307, 323-325, 346, 347, 364-366, 372, 381, 384, 389, 394, 397, 423, 425, 426, 432, 433, 438, 442, 449, 451, 458, 467, 470, 477, 481, 488, 523, 553, 603, 610, 611, 615, 617, 625, 640, 659, 726, 729, 739, 759, 761, 769-774]), Pneumonia/LRTI – 560 cases (146 studies[19, 22, 35, 39, 43, 53, 75-77, 83, 87, 108, 112, 116, 119-122, 124, 126, 128, 129, 131, 133, 139, 141, 142, 148, 149, 154, 155, 157, 160, 165, 168, 169, 174, 175, 185, 190, 191, 194, 199, 204, 205, 211, 217-219, 221, 226, 228, 231, 232, 240, 241, 244, 248, 249, 251, 252, 256, 257, 260, 263, 265, 267, 272-275, 278, 281, 284, 298, 299, 302, 305, 317, 319, 324, 325, 331, 340, 345, 353, 360, 364, 365, 369, 372, 381, 394, 401, 403, 411, 423, 425, 426, 432, 433, 435, 438-440, 442, 451, 455, 458, 467, 470, 477, 487-489, 497, 524, 525, 529, 534, 535, 538, 541, 543, 547, 552, 553, 603, 611, 625, 657, 659, 689, 699, 704, 710, 712, 737, 739, 761, 763, 765, 771-774]

**6c – Non-infectious respiratory manifestations**

Bronchiectasis – 259 cases (52 studies[75, 76, 87, 112, 123, 132, 142, 168, 174, 175, 186, 199, 200, 211, 219, 228, 231, 244, 255, 264, 272, 284, 285, 319, 331, 342, 353, 382, 394, 423, 432, 491, 497, 510, 513, 524, 529, 547, 573, 580, 615, 617, 624, 631, 681, 706, 743, 761, 772, 775-777]), Interstitial lung disease/pulmonary fibrosis/chronic lung disease – 48 cases (18 studies[76, 203, 219, 231, 281, 289, 302, 394, 432, 513, 573, 614, 704, 706, 710, 731, 778, 779]), Pneumothorax – 50 cases (13 studies[91, 175, 240, 281, 408, 473, 493, 513, 706, 712, 773, 775, 780]), Asthma – 17 cases (5 studies[76, 195, 429, 582, 740]), Allergic rhinitis – 9 cases (4 studies[76, 142, 372, 763]), Pneumonitis – 6 cases (6 studies[112, 259, 289, 524, 543, 781]), Obstructive sleep apnoea – 1 case (1 study[429]), Bronchitis – 76 cases (21 studies[43, 83, 108, 111, 175, 232, 238, 278, 305, 310, 323, 347, 369, 381, 489, 541, 552, 573, 615, 699, 711]).

**6e – Malignancy**

– Unspecified malignancy – 262 cases (33 studies[24, 32, 36, 61, 154, 201, 205, 211, 234, 270, 346, 349, 374, 409, 580, 610, 637, 711, 730, 755, 775, 782-793]), Other adenocarcinoma – 14 cases (2 studies[692, 791]), Cerebral malignancy – 16 cases (15 studies[43, 44, 52, 99, 288, 337, 364, 372, 482, 490, 634, 641, 794-796]), Breast cancer – 38 cases (15 studies[24, 28, 35, 36, 44, 52, 55, 66, 69, 98, 175, 245, 337, 363, 424]), Other – 46 cases (35 studies[22, 23, 24 , 35, 44, 61, 66, 80, 154, 166, 208, 233, 262, 283, 287, 333, 341, 346, 407, 408, 462, 473, 498, 626, 659, 663, 797-806]), Dermatofibrosarcoma – 5 cases (5 studies[24, 35, 36, 44, 807]), Gastrointestinal malignancy – 33 cases (25 studies[61, 72, 87, 91, 130, 175, 185, 190, 243, 276, 296, 337, 346, 374, 375, 385, 391, 441, 621, 771, 797, 808-811]), Germ cell tumour – 2 cases (2 studies[154, 462]), Unspecified haematological malignancy - 7 cases (3 studies[52, 357, 449]), Unspecified haemopoietic malignancy – 47 cases (1 study[775]), Hepatic malignancy – 24 cases (19 studies[24, 52, 66, 130, 225, 294, 312, 337, 374, 422, 765, 812-819]), Hodgkin's disease – 175 cases  (60 studies[24, 44, 52, 75, 76, 78, 87, 100, 113, 116Thibaud, 2019 #175350, 133, 150, 182, 204, 209, 252, 255, 278, 304, 310, 337, 355, 369, 370, 378, 397, 458, 551, 614, 625, 626, 668, 676, 699, 712, 765, 769, 771, 776, 779, 783, 791, 795-797, 808, 810, 814, 820-830]), Smooth muscle tumour – 12 cases (8 studies[362, 456, 626, 831-834]), Leukaemia – 308 cases (111 studies[15, 24, 35, 36, 44, 50, 52, 66, 72, 75, 76, 79, 82, 87, 93, 102, 108, 114, 124, 127, 142, 154, 175, 182, 208, 229, 264, 277, 278, 280, 302, 303, 308, 316, 337, 339, 346, 347, 358, 362, 364, 369, 374, 376, 404, 410, 424, 435, 437, 445, 448, 464, 465, 469, 471, 479, 485, 494-496, 508, 529, 530, 536, 537, 551, 562, 574, 575, 611, 614, 626, 636, 699, 704, 712, 743, 765, 783, 791, 796, 797, 823, 825, 826, 831, 835-859]), Unspecified lymphoid malignancy – 92 cases (5 studies[61, 579, 626, 823, 860]), Undefined/other lymphoma – 241 cases (61 studies[24, 44, 52, 61, 66, 88, 90, 92, 93, 108, 137, 142, 148, 155, 169, 175, 209, 250, 264, 271, 275, 278, 294, 311, 374, 382, 420, 421, 429, 438, 484, 491, 510, 513, 551, 570, 626, 660, 690, 699, 712, 738, 743, 771, 783, 791, 830, 848, 861-873]), Lymphoproliferative disorder – 16 cases (5 studies[452, 617, 669, 678, 771]), Non-Hodgkin's lymphoma – 450 cases (89 studies[24, 35, 36, 44, 49, 75, 76, 87, 92, 99, 101, 105, 109, 130, 143, 177, 182, 204, 209, 213, 214, 220, 239, 264, 278, 303, 304, 337, 346, 352, 354, 359, 373, 376, 387, 405, 436, 438, 454, 470, 474, 492, 494, 529, 552, 553, 567, 571, 614, 624, 626, 712, 765, 769, 795-797, 808, 810, 814, 815, 817, 824, 826, 828, 839, 846, 866, 872-892]), Bone malignancy – 2 cases (2 studies[626, 885]), Ovarian malignancy – 6 cases (5 studies[231, 312, 432, 462, 893]), Pancreatic malignancy – 5 cases (5 studies[24, 33, 35, 44, 431]), Renal malignancy – 8 cases (8 studies[61, 66, 154, 577, 813, 817, 894, 895]), Sarcoma – 8 cases (8 studies[16, 61, 209, 419, 500, 770,

[852, 896]), Skin malignancy – 4 cases (4 studies[333, 423, 711, 897]), Unspecified solid malignancy – 50 cases (7 studies[175, 449, 743, 771, 783, 823, 826]), Thyroid malignancy – 20 cases (16 studies[44, 76, 115, 175, 209, 337, 365, 388, 393, 431, 501, 626, 797, 801, 824, 898]).

**7a - Reported numerical values of AFP - Raised AFP – 354 cases (161 studies**[24, 27, 30, 33, 36, 38, 41-43, 47, 48, 52, 54, 59, 61-63, 65, 66, 72, 74, 76, 83, 89, 91, 92, 95, 99-101, 105, 115, 120-124, 128, 129, 132, 134, 135, 138, 148, 150, 165, 168, 184, 193, 195-197, 199, 203, 211, 214, 217, 223, 225, 227, 228, 233, 236, 238, 239, 246-248, 251, 252, 257, 259, 261-266, 268-270, 272, 273, 294, 338, 346, 354, 363, 368, 369, 375, 381-383, 385, 388, 390, 391, 395, 396, 398, 404, 405, 411, 414, 416, 417, 419, 422, 424, 428, 429, 431, 433, 443, 445, 448, 451, 460, 461, 464, 470, 476, 479, 481, 482, 495, 497, 501, 516, 522, 528, 530, 532, 536, 540, 556, 558, 570, 575, 583, 591, 599, 625, 641, 642, 661, 693, 699, 715, 768, 804, 893, 899, 900]

**7c – Endocrine manifestations**

Diabetes – 56 cases (31 studies[24, 33, 49, 52, 77, 115, 119, 142, 178, 190, 201, 204, 211, 347, 364, 372, 409, 410, 449, 636, 655, 659, 704, 747, 768, 860, 897, 908-911]), Low vitamin D – 96 cases (7 studies[636, 653, 771, 912-915]

Hypothyroidism – 12 cases (6 studies[77, 198, 201, 226, 897, 913]), Hyperthyroidism – 2 cases (1 study[449]), Biochemical lipid disorders – 92 cases (11 studies[33, 117, 342, 346, 636, 655, 747, 768, 909, 916, 917])

**7f – Skin manifestations**

Café-au-lait – 94 cases (29 studies[63, 76, 87, 91, 108, 111, 131, 176, 181, 184, 198, 208, 210, 228, 229, 231, 234, 260, 282, 315, 325, 364, 369, 450, 507, 520, 521, 602, 926]), Eczema – 45 cases (5 studies[87, 157, 218, 287, 704]), Granuloma – 114 cases (54 studies [86, 87, 148, 165, 210, 211, 219, 228, 246, 247, 260, 264, 270, 294, 327, 345, 418, 427, 447, 451, 452, 470, 501, 517, 533, 556, 582, 602, 628, 642, 678, 695, 709, 715, 717, 718, 722, 731, 737, 741, 746, 751, 755, 792, 861, 927-935]), Pigmentary abnormalities – 148 cases (33 studies[43, 63, 76, 100, 111, 126, 184, 197, 212, 222, 232, 262, 274, 283, 285, 315, 323, 335, 368, 369, 426, 428, 447, 448,

488, 499 Moreno, 2005 #75802, 529, 534, 602, 603, 921, 936]), Other skin manifestation – 48 cases (22 studies [100, 108, 142, 176, 197, 233, 254, 294, 315, 323, 351, 360, 369, 407, 426, 428, 435, 450, 574, 726, 739, 921]), Infection – 15 cases (6 studies[278, 404, 529, 608, 704, 920]), Warts – 65 cases (12 studies[123, 222, 278, 287, 315, 347, 360, 404, 529, 704, 739, 937])

**7g – Orthopaedic manifestations**

Scoliosis 117 cases (24 studies[52, 71, 76, 79, 106, 118, 126, 168, 175, 180, 190, 225, 231, 240, 288, 366, 372, 385, 449, 706, 726, 738, 739, 938]), Pes cavus – 29 cases (9 studies[17, 53, 109, 213, 225, 287, 335, 345, 472]), Equinus foot deformity – 4 cases (4 studies[213, 237, 347, 403, 404]), Tight Achilles tendon – 26 cases (3 studies[225, 287, 335]).

**8a – Gastrointestinal manifestations**

Gastroesophageal reflux – 8 cases (5 studies[91, 226, 236, 449, 939]), Aspiration – 34 cases (10 studies[39, 181, 240, 242, 408, 706, 711, 712, 771, 940]), Dysphagia – 115 cases (24 studies[52, 76, 83, 108, 109, 121, 142, 166, 175, 191, 230, 240, 269, 284, 288, 408, 449, 590, 639, 653, 654, 711, 712, 941]), Recurrent/chronic diarrhoea – 56 cases (13 studies[53, 75, 99, 124, 132, 142, 157, 180, 197, 523, 603, 704, 942]), Gastrostomy in situ – 96 cases (20 studies[35, 39, 226, 228, 408, 457, 564, 626, 653, 654, 711, 712, 754, 761, 771, 778, 845, 905, 943, 944]).

**8d – Cognition, education and employment** - Degree educated – 11 cases (5 studies[35, 59, 62, 103, 644]), Mainstream school – 5 cases (5 studies[122, 126, 222, 233, 438]), Special school – 8 cases (8 studies[21, 112, 122, 126, 231, 254, 472, 479]), Employed as an adult – 16 cases (5 studies [17, 30, 35, 53, 62]), Living independently as an adult – 2 cases (2 studies[53, 396]), Impaired cognitive function – 123 cases (62 studies)[2, 27, 33, 41, 75, 87, 101, 104, 106, 108, 109, 112, 113, 120, 121, 123, 126, 129, 147, 152, 186, 190, 191, 208, 212, 238, 240, 242, 251, 254, 255, 298-300, 310, 324, 325, 331, 335, 338, 345, 368, 372, 389, 396, 401, 403, 407, 416, 432, 449, 472, 518, 524, 528, 536, 541, 580, 603, 656, 841, 844, 945])

**8e – Cause of death** - 752 causes of death in 687 cases (168 studies[24, 28, 33, 35, 36, 43, 49, 52, 57, 61, 66, 72, 75, 76, 78-80, 87, 90-93, 99, 108, 114, 116, 121, 124, 128, 131-134, 139, 142, 155, 158, 160, 166, 177, 185, 190, 201, 204, 208, 211, 220, 221, 225, 229, 240, 242, 250, 255, 256, 264, 276, 278, 287 #79876, 290, 294, 296, 302, 305, 310, 312, 317, 319, 337, 339, 341, 345-347, 352, 354, 355, 358, 370, 373, 376, 378, 382, 385, 391, 397, 400, 407, 410, 427, 431, 432, 434, 438, 442, 445, 448, 449, 452, 457, 458, 464, 467, 474, 483, 490, 495, 498, 510, 513, 529, 538, 562, 567, 569, 570, 574, 575, 577, 579, 603, 614, 615, 624, 626, 657, 668, 669, 678, 690, 704, 711, 712, 730, 738, 765, 770, 771, 776, 778, 779, 781, 796, 797, 801, 817, 818, 822, 825, 827, 830, 840, 846, 849, 860, 861, 870, 872, 875, 880, 886, 887, 891, 946, 950, 951, 955, 956]). **Unknown cause of death, but death reported - 1021 cases (84 studies**[24, 36, 61, 62, 70, 75, 78, 88, 90, 127, 130, 139, 154, 156, 162-164, 175, 187, 205, 231, 270, 275, 298, 304, 327, 337, 345, 346, 350, 364, 374, 394, 400, 407-409, 424, 434, 449, 473, 489, 563, 566, 576, 610, 615, 626, 638, 660, 676, 681, 687, 706, 743, 765, 795, 797-799, 801, 828, 831, 839, 851, 864, 871, 872, 888, 890, 894, 896, 947-949, 952-954, 957-960])

Supplementary figure 1 - Incorrect, incomplete and missed diagnoses – Cerebral palsy – 74 cases (26 studies[16, 59, 76, 103, 108, 111, 150, 166, 177, 214, 219, 222, 236, 242, 287, 310, 314, 372, 384, 404, 438, 469, 471, 488, 573, 592]), Gait abnormality – 10 cases (6 studies[86, 206, 387, 478, 518, 592]), Dystonia – 7 cases (4 studies[28, 30, 47, 546]), Immune dysfunction – 10 cases (8 studies[24, 128, 203, 228, 373, 416, 592]), Other – 22 cases (13 studies[71, 76, 86, 208, 211, 228, 396, 404, 433, 476, 492, 518, 592]).

Supplementary figure 2 – Breakdown of recurrent infections

See recurrent infections (figure 6b) references.

Supplementary figure 3 – Presenting symptoms of Hodgkin's lymphoma

See malignancy (6e) references.

Supplementary figure 4 – Presenting symptoms of non-Hodgkin's lymphoma

See malignancy (6e) references.

Supplementary figure 5 - Presenting symptoms of leukaemia

See malignancy (6e) references.

Supplementary figure 6 – Other telangiectasia

See other telangiectasia in text references.

**Supplementary figure 7 - Indication for gastrostomy** – Underweight – 21 cases (4 studies[653, 761, 771, 944]), Swallowing difficulties – 22 cases (3 studies[653, 712, 771]), Nutritional support – 32 cases (3 studies[711, 712, 778]), Malignancy – 1 cases (1 study[626]), Palliative – 2 cases (2 studies[712, 771]), Recurrent lung infection – 2 cases (1 study[712]), Weight loss – 3 cases (1 study[712]), FVC<20% - 1 cases (1 study[712]), Unknown indication – 37 cases (14 studies[35, 39, 226, 228, 408, 457, 564, 654, 754, 767, 845, 905, 943, 944]).

**Supplementary figure 8 - Other medical problems – 137 studies** [17, 24, 33, 52, 53, 57, 71, 76, 77, 107, 108, 113, 116, 121, 124, 126, 137, 157, 160, 176, 190, 196, 197, 211, 213, 214, 221, 224, 226, 228, 231, 236, 237, 240, 243, 249, 257, 259, 264, 266, 267, 277, 284, 289, 294, 295, 315, 334, 336, 346, 347, 359, 364, 366, 368, 369, 379, 380, 382-385, 408, 410, 413, 414, 428, 431-433, 443, 445, 448-450, 455, 463, 466-468, 478, 482, 483, 498, 502, 518, 523, 529, 547, 552, 562, 563, 573, 581, 586, 590, 602, 618, 629, 630, 640, 657, 659, 661, 686, 704, 712, 717, 738, 744, 752, 768, 774, 781, 831, 837, 845, 859, 883, 895, 896, 911, 913, 927, 934, 936, 941, 962-970]).

**Supplementary figure 9 – Delayed neurological development in early life.**

Sitting – 30 cases (23 studies[108, 111, 126, 129, 139, 222, 225, 262, 270, 310, 324, 325, 331, 366, 379, 403, 404, 406, 415, 461, 479, 518, 572]), Walking – 94 cases (62 studies[33, 34, 54, 63, 71, 74, 84, 108, 111, 113, 126, 129, 135, 150, 184, 194, 212, 222, 225, 231, 236, 242, 248, 261-263, 265, 269, 270, 276, 288, 290, 305, 310, 319, 324, 325, 331, 340, 354, 372, 379, 380, 387, 400, 401, 403, 404, 406, 407, 415, 433, 458, 472, 479, 489, 513, 518, 538, 541, 572, 643]), First words – 7 cases (6 studies[231, 288, 324, 325, 331, 572]).
