# Supplementary figures and images for: The natural history of ataxia-telangiectasia (A-T): A systematic review
Source: PLoS One. 2022 Mar 15;17(3):e0264177. doi: 10.1371/journal.pone.0264177 (PMC9049793; doi:10.1371/journal.pone.0264177)

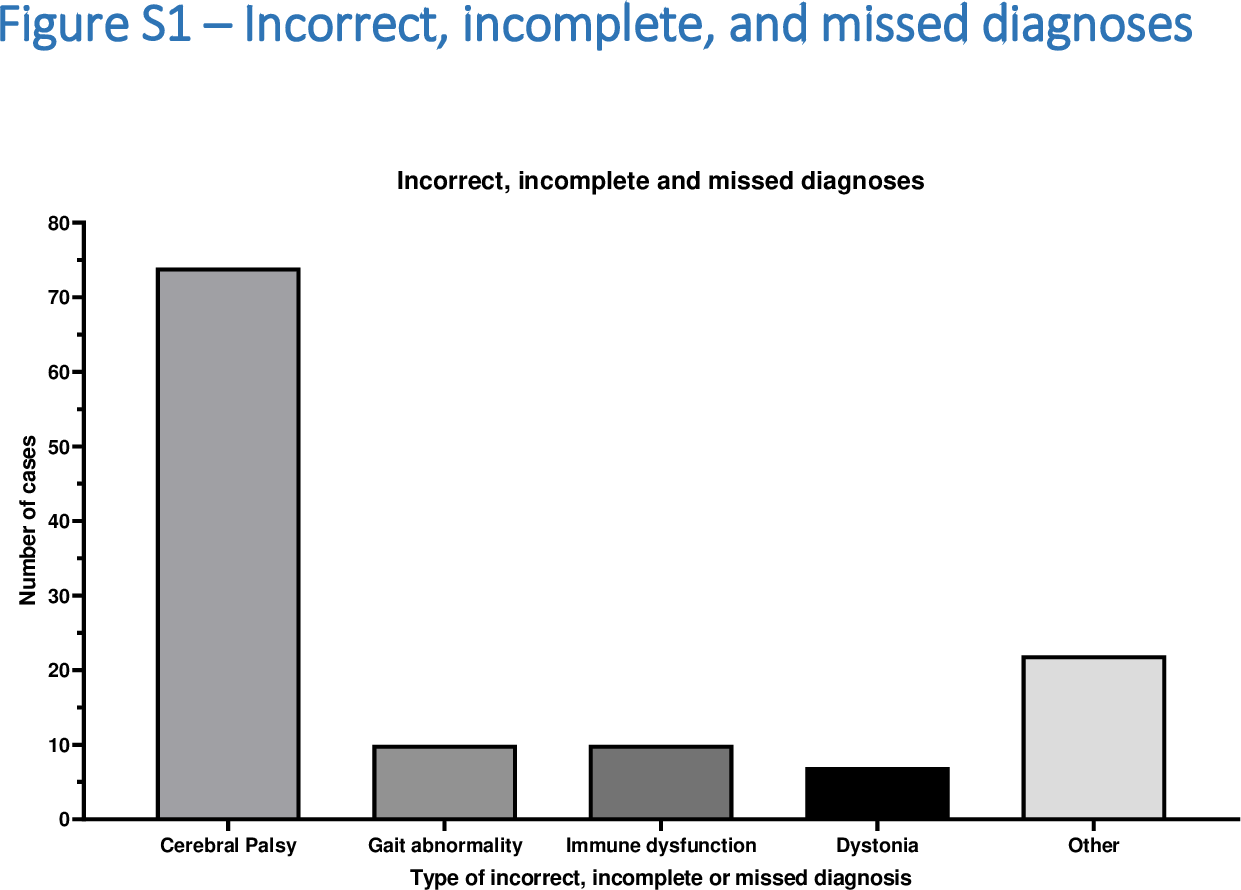

Supplement: S1 Fig — (TIF) [file pone.0264177.s006.tif]

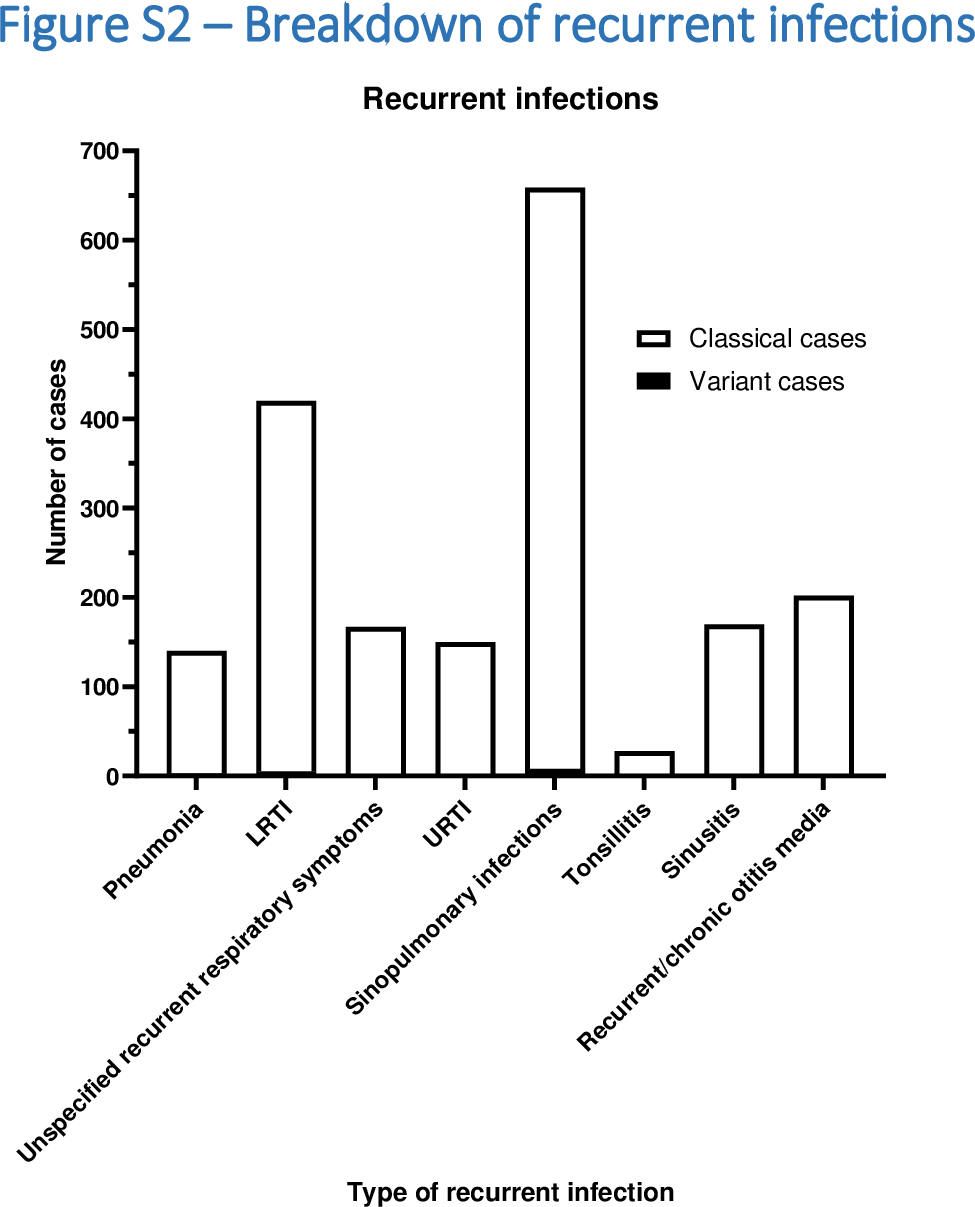

Supplement: S2 Fig — (TIF) [file pone.0264177.s007.tif]

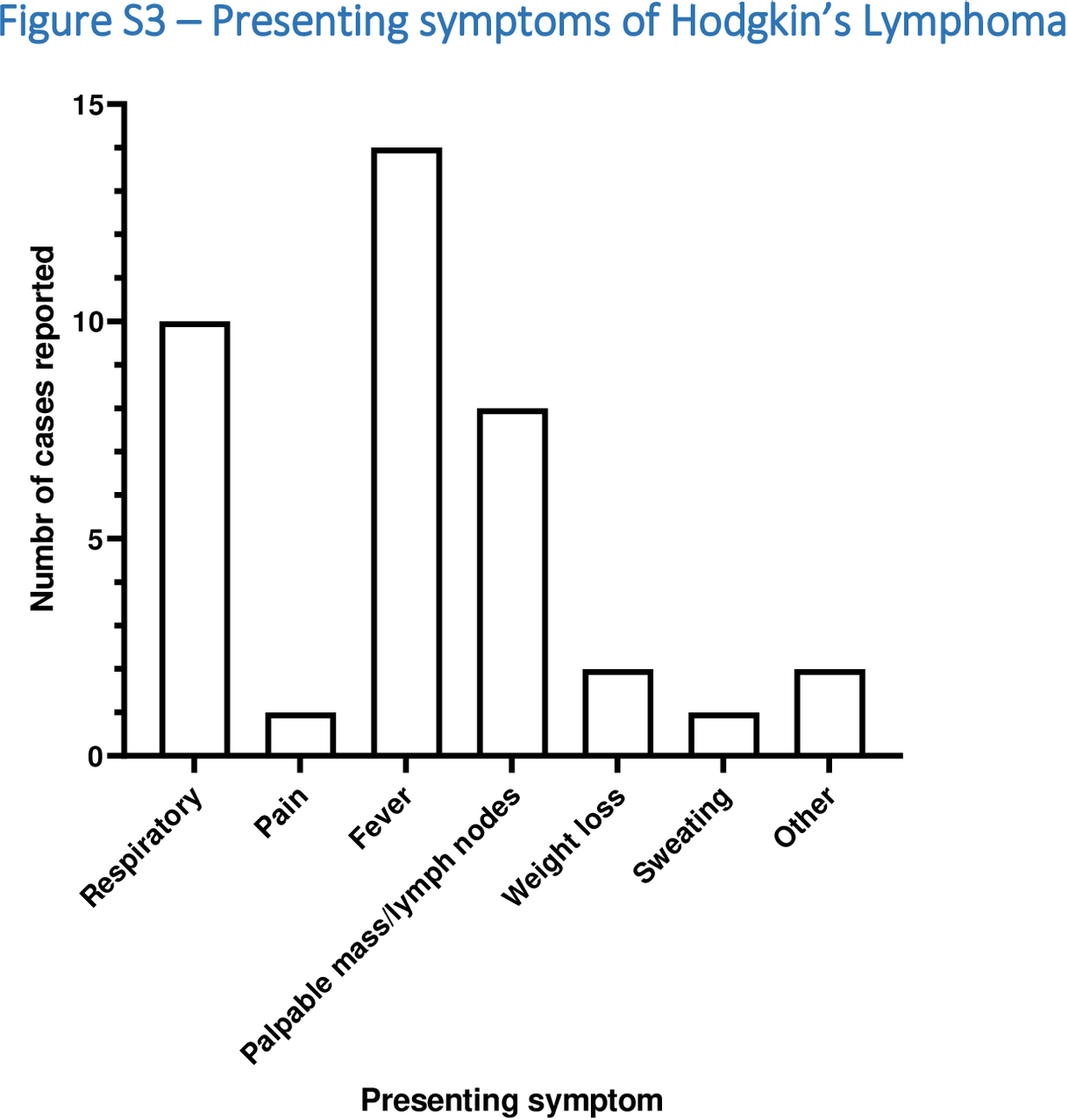

Supplement: S3 Fig — (TIF) [file pone.0264177.s008.tif]

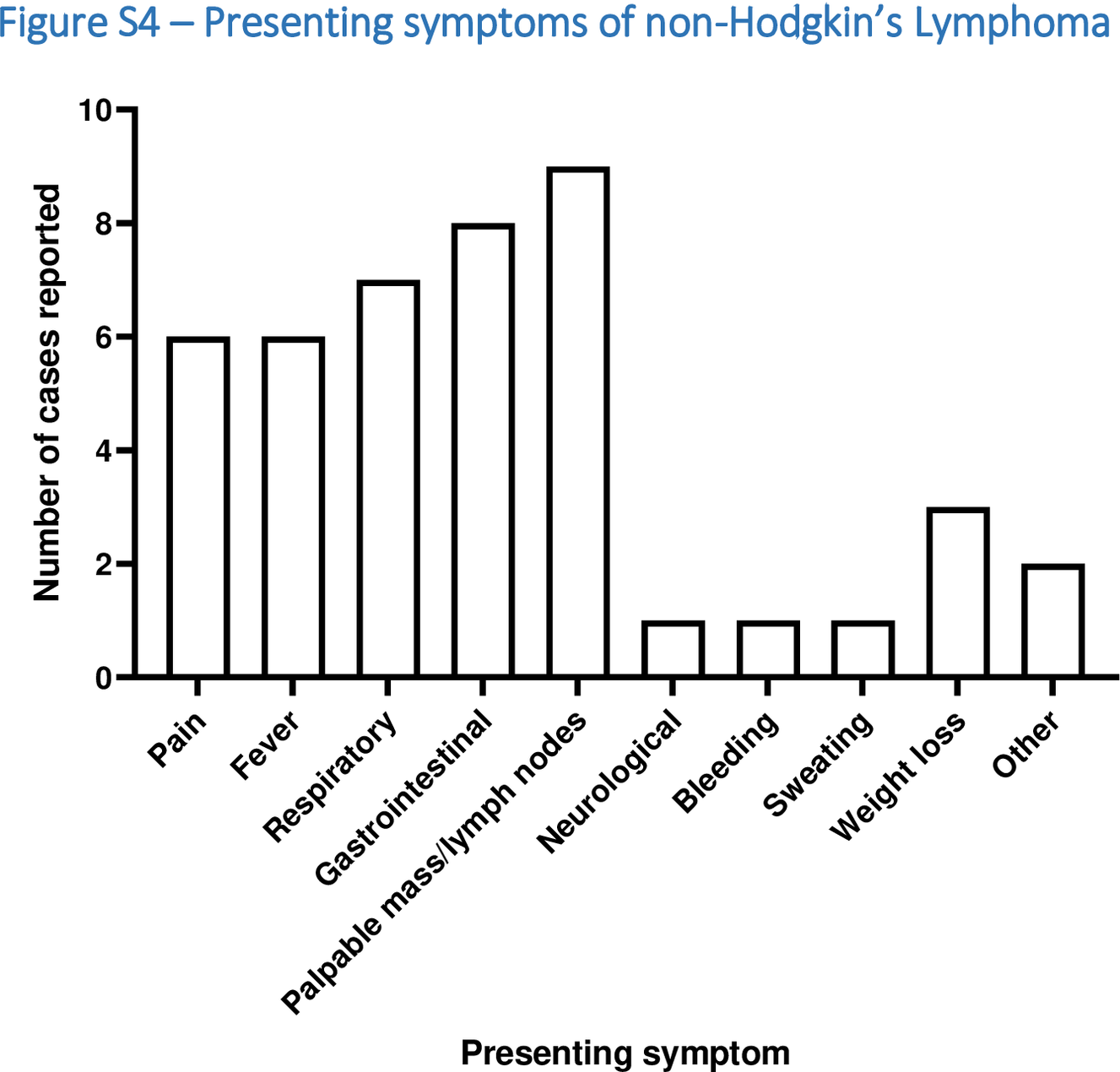

Supplement: S4 Fig — (TIF) [file pone.0264177.s009.tif]

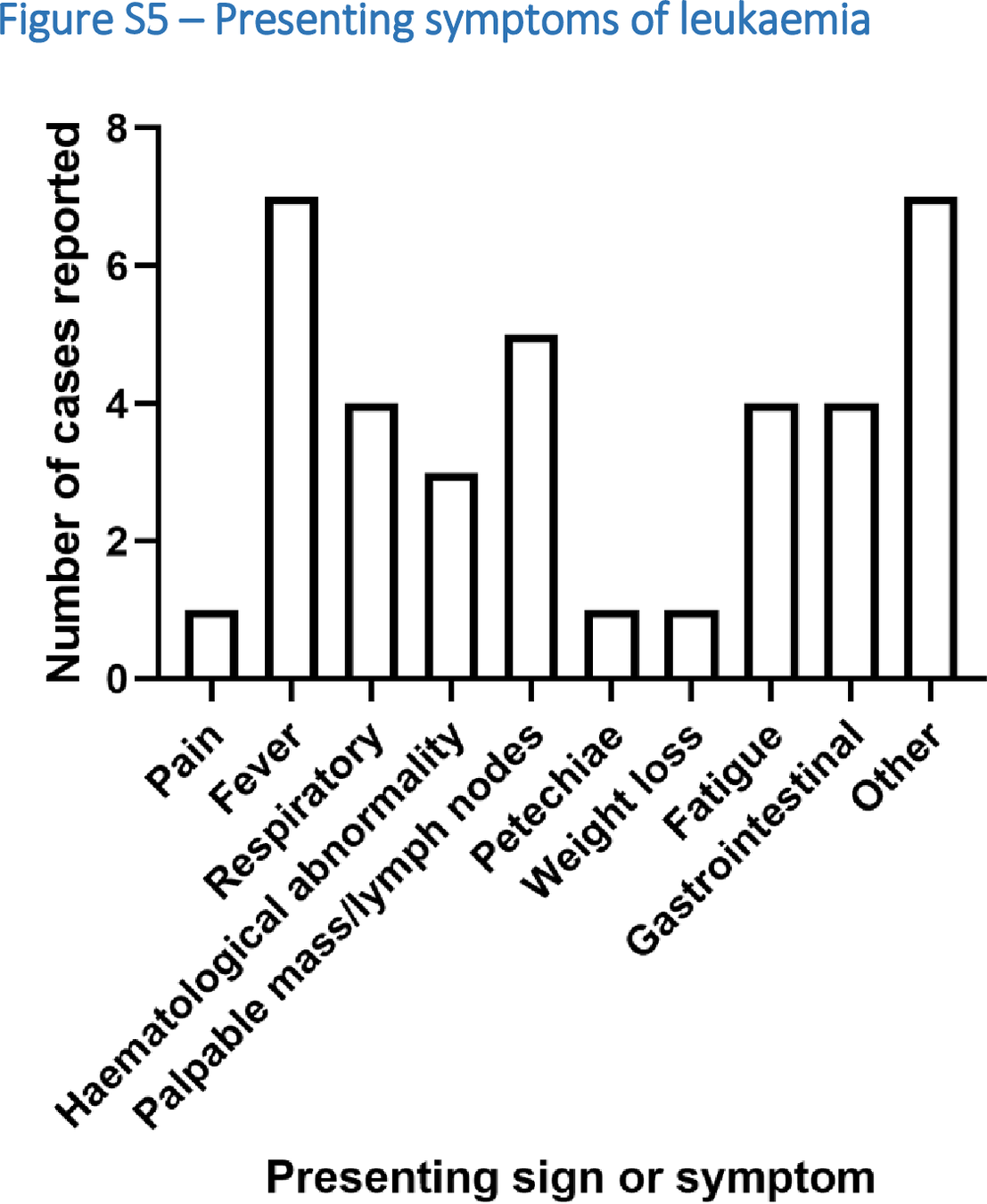

Supplement: S5 Fig — (TIF) [file pone.0264177.s010.tif]

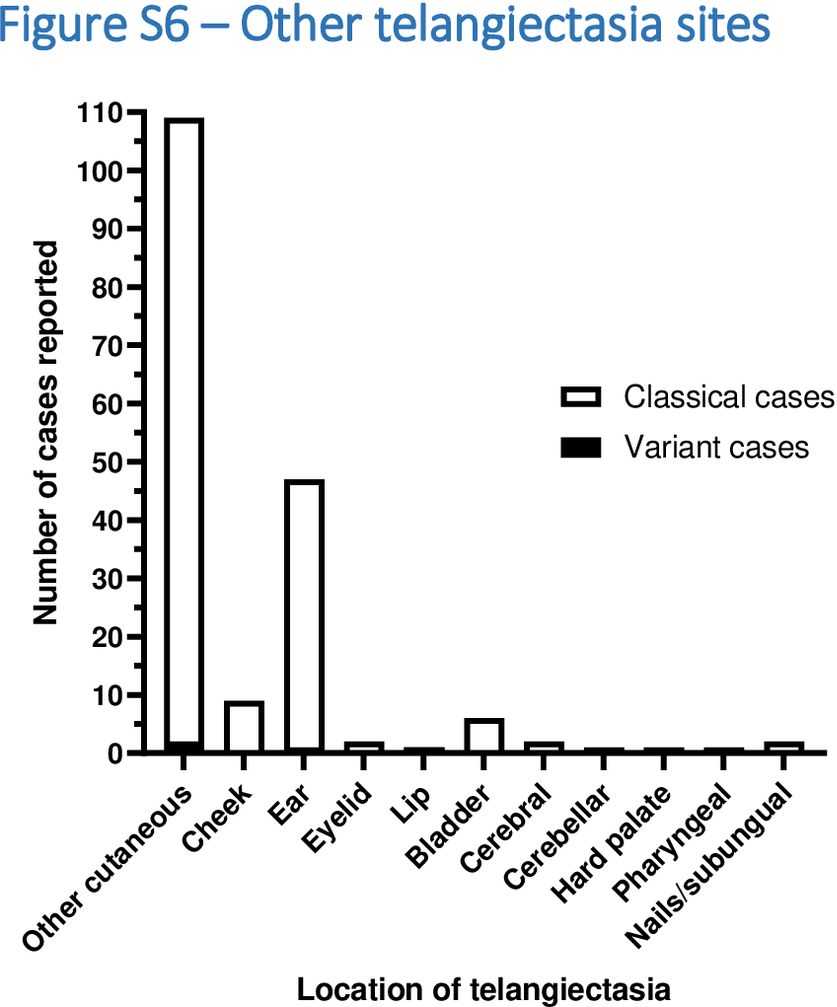

Supplement: S6 Fig — (TIF) [file pone.0264177.s011.tif]

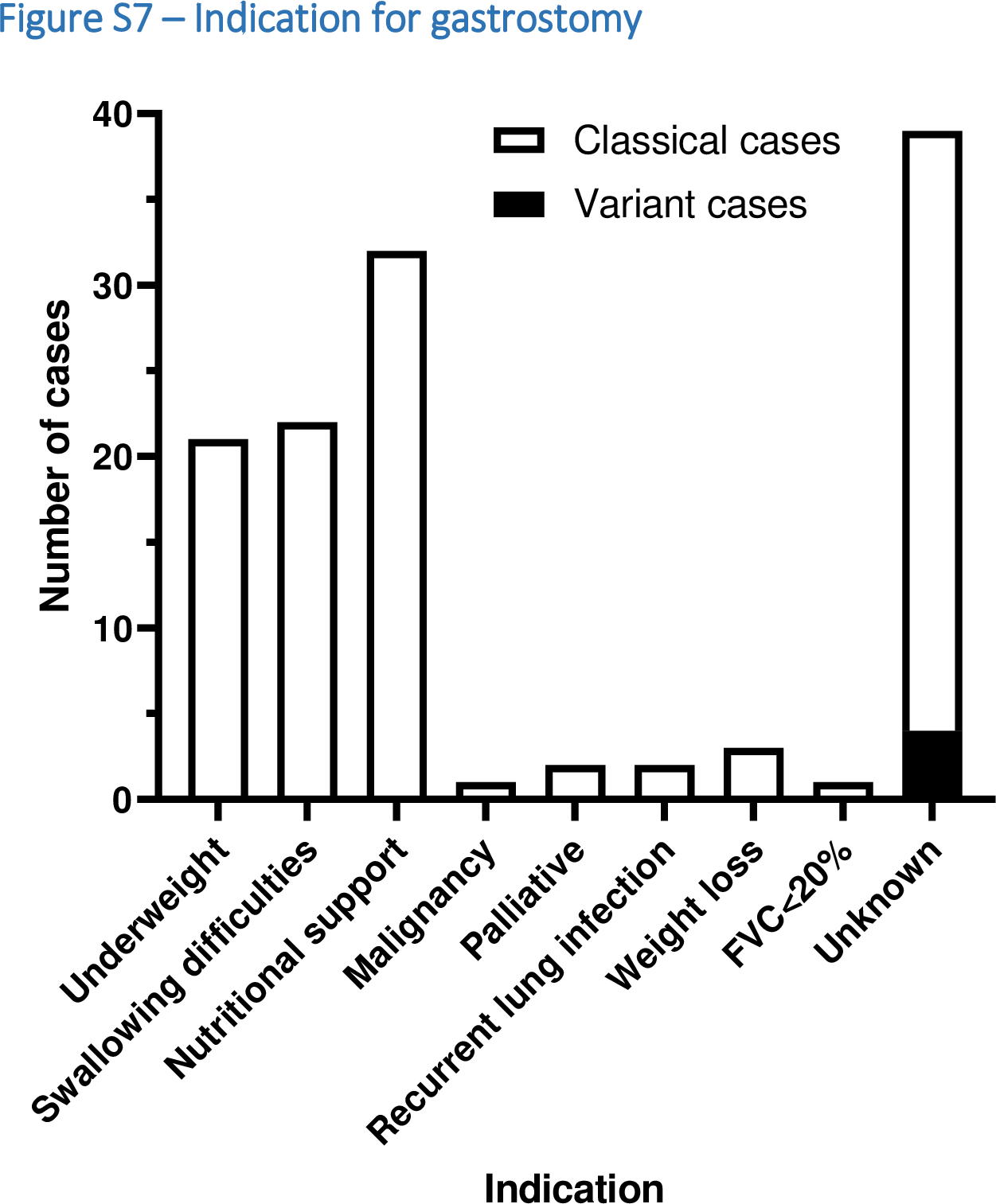

Supplement: S7 Fig — (TIF) [file pone.0264177.s012.tif]

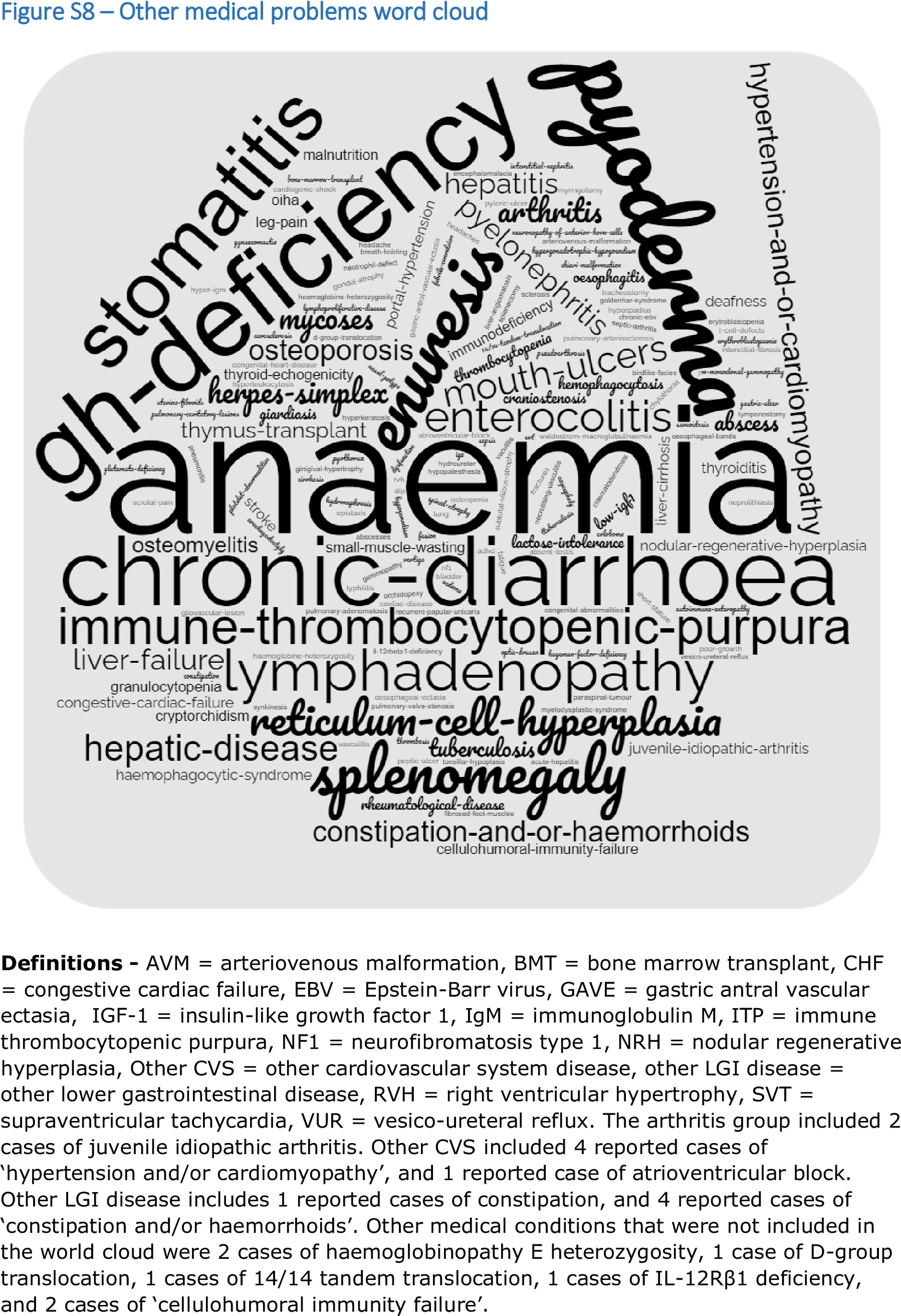

Supplement: S8 Fig — (TIF) [file pone.0264177.s013.tif]

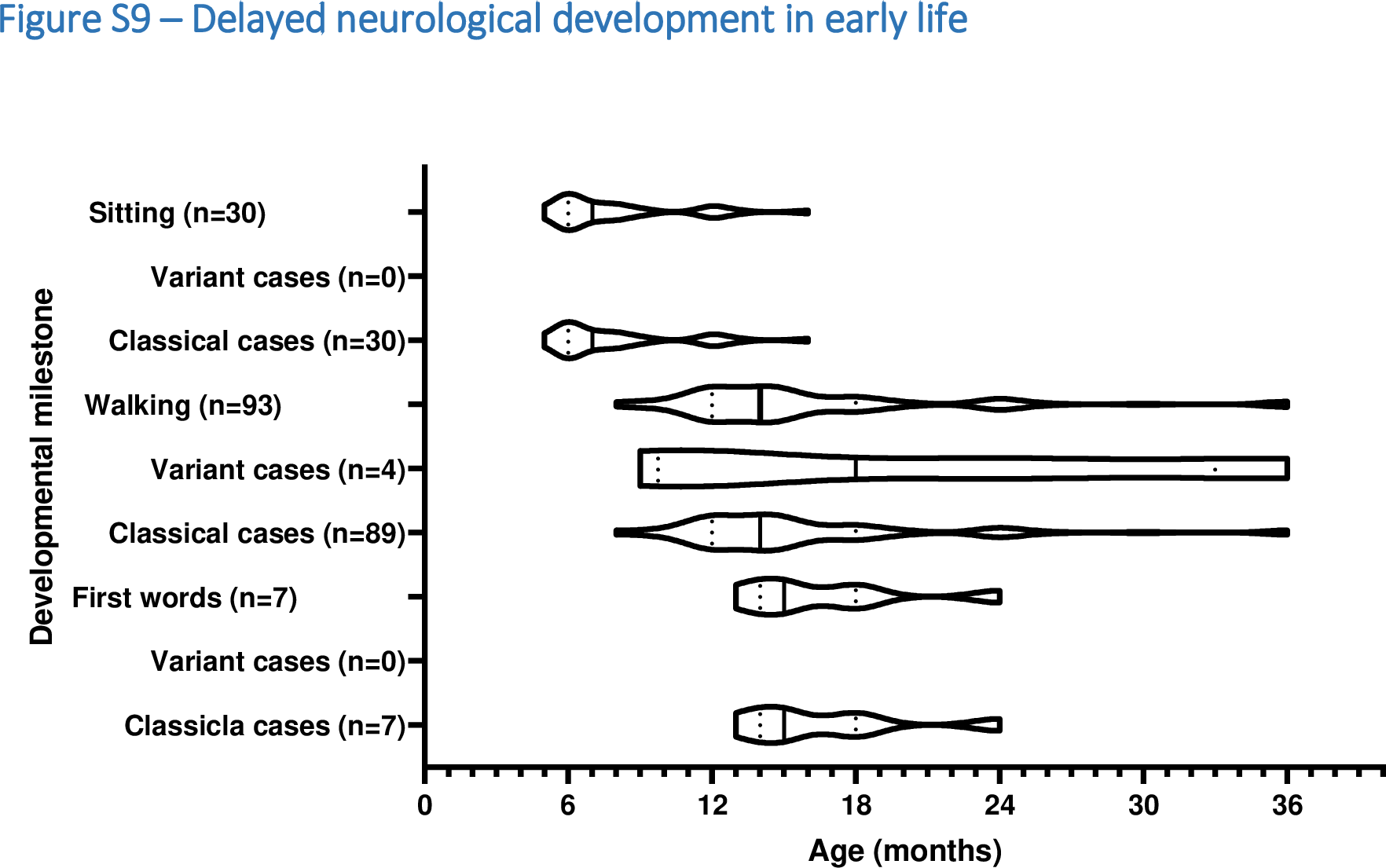

Supplement: S9 Fig — (TIF) [file pone.0264177.s014.tif]
