# Supplementary material for: The natural history of ataxia-telangiectasia (A-T): A systematic review
Source: PLoS One. 2022 Mar 15;17(3):e0264177. doi: 10.1371/journal.pone.0264177 (PMC9049793; doi:10.1371/journal.pone.0264177)
Supplement: Attachment — Submitted filename: Recenze_AT.docx [file pone.0264177.s015.docx]

These are few points and recommendations:

**1. There are many typos in the manuscript**, please, check this carefully (or use an editor in word). Few examples are given below:

p.9,	l.37:	featues

l.39:	demonstate

p.10,	l.58:	progressinve

l.59:	leuraemia

l.59:	logivity

There are many more, but I only searched through first pages of the manuscript, as there are many.

**2. Accurate numbers in the text**

For example description for Figure is not correct:

p.16,	l.167:	The search yielded 14622 titles and abstracts (Figure 1). After removal of 180268 duplicates 168 and exclusion of 13459 articles by review of title and abstract, 1163 full text articles were 169 reviewed.

Instead of 14622 should be 194890?

p.16	l.178:	Age at inclusion was median 178 (IQR) (n=1486) 144.0 months (84.0-240.0).

Should be median age at inclusion?

**3. Reference source not found**

There are many missing references, please, check this more carefully. I am giving few examples, but it was just not possible to copy and paste all the missing references:

p-16	l.183:	There were fewer reports from Africa, parts of South America and the Middle East (Error! Reference source not found. and Error! Reference source not found.).

l.204:	reported an age of

l.223:	Error! Reference source not found

l.227: nystagmus, and saccades (Error! Reference source not found.6d). 17 further cases (1

228 study[461])

l.233:	(Error!

234 Reference source not found.7a). Several cases had progression of the reflexes from

l.245: Included studies reported a wide range of movement disorders (Error! Reference source

246 not found.. D

**l.266-268:** Reported immunoglobulin levels are shown in (**Error! Reference source not found.**7e).

267 699 cases (124 studies) reported the use of immunoglobulin replacement therapy (**Error!**

268 **Reference source not found.**

271 233 cases (43 studies) reported the start of use of prophylactic antibiotics (**Error! Reference**

272 **source not found.** 7f and 8a)

4.	l.259:	Electro myo-graphy (EMG)

Should be Electromyography ?

5.	l.329: 4 cases

l.338: 7 cases

Should be:

Four cases..; Seven cases (beginning of the sentence)

6.	l.446: We are not aware of a routine screening protocol for malignancy in people

446 with A-T,

This is very significant, there are protocols, but maybe only in local recommendations? This should be clarified. In our country there is a recommendation:

MUDr. Věra Krutílková; prof. MUDr. Tomáš Eckschlager, CSc.

Klin Onkol 2009; 22(Suppl 1): 45-49. The Survey of Syndromes with the Risk of Cancer in Children Age

The protocol is very important and definitely should be included in the paper.

7.	l.477:	Despite our comprehensive literature seach and review, we did not find population-based 478 studies and were unable to determine the prevalence of A-T

8. Figure 4

For example there is only one patient listed form the Czech Republic and you cited two Czech papers (ref. 125 and 248.)

However, the reference 248 listed 12 patients from the Czech rep…

There are some inconsistencies…

Soukupova, J., P. Pohlreich, and E. Seemanova, *Characterisation of ATM mutations in Slavic Ataxia telangiectasia patients.*

1007 NeuroMolecular Medicine, 2011. **13**(3): p. 204-11.
