# Supplementary material for: The natural history of ataxia-telangiectasia (A-T): A systematic review
Source: PLoS One. 2022 Mar 15;17(3):e0264177. doi: 10.1371/journal.pone.0264177 (PMC9049793; doi:10.1371/journal.pone.0264177)
Supplement: Attachment — Submitted filename: letter to editor v3 31-10-2021.docx [file pone.0264177.s016.docx]

Dear Dr Chen,

Thank you for reviewing our manuscript and sending us the reviewer comments. We have updated the data to 19^th^ August 2021 and made changes to the manuscript in response to the reviewer comments. Our response to the comments is below:

| **Line number** | **Comment** | **Action** |
| --- | --- | --- |
|  | Please confirm that you have included all items recommended in the PRISMA checklist including:  - the full electronic search strategy used to identify studies with all search terms and limits for at least one database. | The search strategy is attached as a supplementary file |
|  | Update search and analysis to include studies published since December 2019 | Searches have been updated to 19th August 2021 |
|  | We note that Figures 3 and 4 in your submission contains map images which may be copyrighted. All PLOS content is published under the Creative Commons Attribution License (CC BY 4.0), which means that the manuscript, images, and Supporting Information files will be freely available online, and any third party is permitted to access, download, copy, distribute, and use these materials in any way, even commercially, with proper attribution. We require you to either (1) present written permission from the copyright holder to publish these figures specifically under the CC BY 4.0 license, or (2) remove the figures from your submission: | We have removed the images from the manuscript |
|  | We note that this manuscript is a systematic review or meta-analysis; our author guidelines therefore require that you use PRISMA guidance to help improve reporting quality of this type of study. Please upload copies of the completed PRISMA checklist as Supporting Information with a file name “PRISMA checklist". | We have included the PRISMA checklist |
| 167 | The search yielded 14622 titles and abstracts (Figure 1). After removal of 180268 duplicates and exclusion of 13459 articles by review of title and abstract, 1163 full text articles were reviewed. Results do not sum up in paragraph one of results, in particular in the first sentence. Has to be checked | This has been corrected and updated with the new results |
| 223 and others | References sources are missing on too many occasions in the text. This aspect is of course crucial in he context of a review article and requires a thorough reassessment.  One example Line 223: Error! Reference source not found; line | We have ensured that references are now correctly embedded |
|  | Authors confirm the increased risk of malignancies. For example: “ lymphoma and leukaemia were the most common malignancies reported”. More information regarding mean age of onset should be described in the main body of the text, as an important clinical information element. Age of onset is mentioned as a secondary endpoint in the methods, so this information should be available. | Data on age of diagnosis of non-Hodgkin's lymphoma, Hodgkin's disease and leukaemia have been added to the main text |
| The paragraph on EMG | EMG description is limited to normal vs abnormal criteria which is very vague. Authors should try to provide more detailed information on sensory nerve conduction vs CMAP, velocities etc… | Where relevant data are available we have now presented these data. Since this is a systematic review we are limited to the data reported. |
| 427 | ”As A-T is a rare disease, it is not unusual for the condition to be misdiagnosed. We found that, most often, A-T was mis-labelled as cerebral palsy (CP)”. There are several forms of CP. Do authors refer to the Ataxic form? If yes this should be added. | This statement has been added |
| table 3 | typos: “ataxia-telangiectasia” | This has been corrected |
| Title | I would suggest to not entitle this work a scoping review but much more a systematic review. The authors conducted a structured and extensive literature search and extraction of full texts as well as data in a systematic way. Moreover, the rated each study where applicable by the recommended risk of bias tools. You should then include a paragraph with the information on the level of evidence of the included publications rated by one of the established LoE tools. | All the data are observational and therefore we feel it is difficult to apply the GRADE system. All the studies would be GRADE 4 or 6. We have used an assessment of bias for case-control and cohort studies. |
| Methods | The authors describe the search terms. I would like to suggest to also include a paragraph on the initial PICO question that informed the literature search. | We have added this to the methods |
| 466 | Vit D levels were normal in a third of reported data sets - due to supplementation or without any supplementation? Should VitD be supplemented in any case? | Unfortunately these data are not clearly reported in the literature |
|  | I would like to suggest to give a short outlook on overall management in terms of a multimodal interdisciplinary approach. You may include an outlook on future novel therapeutic approaches, if applicable (i.e. any kind of molecular/genetic therapy in the pipeline?). | We feel that this is out the scope of the review. |
| References | Some references are not correctly embedded. Please check troughout the manuscript and reference list | We have ensured that references are now correctly embedded |
|  | I would certainly recommend that phenotype - genotype correlation is included in the paper. In line with this, authors should define "classical" and "variant" phenotype based on the type of the mutations. Or at least, a comparison should be provided. It is not sufficient to state that all the cases without detailed information were claswsified as "classical". | We appreciate that there may be inaccuracies within our classification. However, where there is no reported mutation, or ATM kinase activity we are unable to check the authors description in further detail, and therefore feel the most appropriate method to not skew the data, given the small % of variant A-T cases, is to include them as classical cases. |
| 37 | featues | This has been corrected |
| 39 | demonstate | This has been corrected |
| 58 | progressinve | This has been corrected |
| 59 | leuraemia | This has been corrected |
| 59 | logivity | This has been corrected |
|  | Other typos | These have been corrected |
| 167 | The search yielded 14622 titles and abstracts (Figure 1). After removal of 180268 duplicates 168 and exclusion of 13459 articles by review of title and abstract, 1163 full text articles were 169 reviewed.  Instead of 14622 should be 194890? | This has been corrected and updated with the new results |
| 178 | Age at inclusion was median 178 (IQR) (n=1486) 144.0 months (84.0-240.0). Should be median age at inclusion? | This sentence has been reworded |
|  | There are many missing references, please, check this more carefully. I am giving few examples, but it was just not possible to copy and paste all the missing references:p-16 l.183: There were fewer reports from Africa, parts of South America and the Middle East (Error! Reference source not found. and Error! Reference source not found.).   l.204: reported an age of  l.223: Error! Reference source not found   l.227: nystagmus, and saccades (Error! Reference source not found.6d). 17 further cases (1 228 study[461])    l.233: (Error! 234 Reference source not found.7a). Several cases had progression of the reflexes from   l.245: Included studies reported a wide range of movement disorders (Error! Reference source 246 not found.. D  l.266-268: Reported immunoglobulin levels are shown in (Error! Reference source not found.7e). 267 699 cases (124 studies) reported the use of immunoglobulin replacement therapy (Error! 268 Reference source not found.   271 233 cases (43 studies) reported the start of use of prophylactic antibiotics (Error! Reference 272 source not found. 7f and 8a) | We have ensured that references are now correctly embedded |
| 259 | Electro myo-graphy (EMG) Should be Electromyography ? | This has been changed |
| 329 and 338 | l.329: 4 cases  l.338: 7 cases  Should be: Four cases..; Seven cases (beginning of the sentence) | These have been corrected |
| 446 | We are not aware of a routine screening protocol for malignancy in people 446 with A-T,  This is very significant, there are protocols, but maybe only in local recommendations? This should be clarified. In our country there is a recommendation:  MUDr. Věra Krutílková; prof. MUDr. Tomáš Eckschlager, CSc. Klin Onkol 2009; 22(Suppl 1): 45-49. The Survey of Syndromes with the Risk of Cancer in Children Age  The protocol is very important and definitely should be included in the paper. | There is no available protocol in the UK. We were unable to obtain a copy of this manuscript in English, including by contacting the corresponding author. We have amended the statement in the discussion |
| Figure 4 | For example there is only one patient listed form the Czech Republic and you cited two Czech papers (ref. 125 and 248.)  However, the reference 248 listed 12 patients from the Czech rep… There are some inconsistencies…  Soukupova, J., P. Pohlreich, and E. Seemanova, Characterisation of ATM mutations in Slavic Ataxia telangiectasia patients. 1007 NeuroMolecular Medicine, 2011. 13(3): p. 204-11. | The country of this paper has been amended. |

Thank you again for the opportunity of improving our paper.

With best wishes

Yours sincerely

William

Dr W P Whitehouse

Honorary (Clinical) Associate Professor

School of Medicine

University of Nottingham, UK

44 (0) 7850 003 927
