# Supplementary material for: The natural history of ataxia-telangiectasia (A-T): A systematic review
Source: PLoS One. 2022 Mar 15;17(3):e0264177. doi: 10.1371/journal.pone.0264177 (PMC9049793; doi:10.1371/journal.pone.0264177)
Supplement: Attachment — Submitted filename: A-T Systematic review Resub response to reviewer 26-01-2022.docx [file pone.0264177.s017.docx]

Response to reviewer

Thank you for your review. We have considered carefully the 3 minor comments made:

*1. “Line 218: “The median delay in diagnosis (n=17) was 0.0 i.e., diagnosed at first presentation but there was wide variation with a range of 0.0-312.0 (IQR, 0.0-43.0) months.” This sentence is hard to understand. Please edit it.”*

We have edited this and tried to clarify it. We added: "Most reported cases were diagnosed without any delay, however a minority were diagnosed late: the median delay in diagnosis (n=17) was 0.0 i.e., diagnosed at first presentation but there was wide variation with a range of 0.0-312.0 (IQR, 0.0-43.0) months.” This is in the Manuscript and in the track changes version resubmitted.

2. *“The subtitle “Cerebellar signs” in Line 264 partially overlaps with the subtitle “Ataxia and Mobility” in Line 230. Please consider integrating these two sections.”*

We are grateful to Tzu-Pu Chang for their comments, and we agree that there is overlap between the "Cerebellar signs" and "Ataxia & Mobility". However, we feel it important not to assume that the mobility problems or even ataxia is always cerebellar ataxia, as children with A-T can have ataxia and mobility impairments caused by frequent myoclonus, chorea, and tremors, as well as neuropathic weakness. So, we believe it is better to report them separately, based on the terminology used in the papers we reviewed.

*3. “Line 430: in Discussion, the authors said “In keeping with the existing view, we found that the median reported age of wheelchair requirement is 10 years.” But I cannot find this statement in the results.”*

Figure 4b shows all reported age data for cerebellar gait ataxia, truncal ataxia, limb ataxia and mobility, including “Wheelchair-bound Classical (n=109)” showing the median age for onset 120 months (10 years).

We hope that this clarification is helpful.
